# Comprehensive and evolutionary analysis of *Spodoptera litura*-inducible Cytochrome P450 monooxygenase gene family in *Glycine max* elucidate their role in defense

**DOI:** 10.3389/fpls.2023.1221526

**Published:** 2023-11-02

**Authors:** Manisha Yadav, Ruby Panwar, Anjana Rustagi, Amrita Chakraborty, Amit Roy, Indrakant K. Singh, Archana Singh

**Affiliations:** ^1^ Department of Botany, Hansraj College, University of Delhi, Delhi, India; ^2^ J C Bose Center for Plant Genomics, Hansraj College, University of Delhi, Delhi, India; ^3^ Department of Botany, Gargi College, University of Delhi, Delhi, India; ^4^ EVA 4.0 Unit, Faculty of Forestry and Wood Sciences, Czech University of Life Sciences Prague, Prague, Czechia; ^5^ Forest Molecular Entomology Lab, EXTEMIT-K, EVA 4.0, Faculty of Forestry and Wood Sciences, Czech University of Life Sciences Prague, Prague, Czechia; ^6^ Molecular Biology Research Lab, Department of Zoology, Deshbandhu College, University of Delhi, New Delhi, India; ^7^ Department of Plant Molecular Biology, University of Delhi, New Delhi, India

**Keywords:** *Glycine max*, *Spodoptera litura*, evolutionary analysis, gene expression analysis, plant defense, herbivory

## Abstract

Plants being sessile organisms and lacking both circulating phagocytic cells and somatic adaptive immune response, have thrived on various defense mechanisms to fend off insect pests and invasion of pathogens. CYP450s are the versatile enzymes, which thwart plants against insect pests by ubiquitous biosynthesis of phytohormones, antioxidants, and secondary metabolites, utilizing them as feeding deterrents and direct toxins. Therefore, a comprehensive analysis of biotic stress-responsive CYPs from *Glycine max* was performed to ascertain their function against *S. litura*-infestation. Phylogenetic analysis and evolutionary studies on conserved domains and motifs disclosed the evolutionary correspondence of these *GmCYPs* with already characterized members of the CYP450 superfamily and close relatedness to *Medicago truncatula.* These *GmCYPs* were mapped on 13 chromosomes; they possess 1-8 exons; they have evolved due to duplication and are localized in endoplasmic reticulumn. Further, identification of methyl-jasmonate, salicylic acid, defense responsive and flavonoid biosynthesis regulating *cis*-acting elements, their interaction with biotic stress regulating proteins and their differential expression in diverse types of tissues, and during herbivory, depicted their responsiveness to biotic stress. Three-dimensional homology modelling of *GmCYPs*, docking with heme cofactor required for their catalytic activity and enzyme-substrate interactions were performed to understand the functional mechanism of their action. Moreover, to gain insight into their involvement in plant defense, gene expression analysis was evaluated, which revealed differential expression of 11 *GmCYPs* upon *S. litura*-infestation, 12 *GmCYPs* on wounding while foliar spray of ethylene, methyl-jasmonate and salicylic acid differentially regulated 11 *GmCYPs*, 6 *GmCYPs*, and 10 *GmCYPs* respectively. Our study comprehensively analysed the underlying mechanism of *GmCYPs* function during *S. litura*-infestation, which can be further utilized for functional characterization to develop new strategies for enhancing soybean resistance to insect pests.

## 1 Introduction

Cytochrome P450s (CYPs) are a diverse and substantial group of monooxygenases found in all kingdoms, including bacteria, fungi, plants and animals (Nelson, 2009). A wide variety of both large and small compounds are included in the substrate spectrum of CYPs. CYPs are named P450-containing enzymes as they often function as final electron acceptors, i.e. terminal oxidase in electron transfer chains (Nelson et al., 1996, Nelson et al., 2006). Empirical evidence suggested that CYPs harbour multiple conserved motifs: an oxygen-binding I-helix (A/G)GX(E/D)T(T/S)], a C-terminus heme-binding site (FXXGXRXCXG), K-helix consensus (EXXR) and PXRX conserved motif. The K-helix and PXRF motif ensures the stability of CYPs fundamental structure by locking the heme pocket into its position (Werck-Reichhart and Feyereisen, 2000).

During the process of evolution, plants have acquired enormous CYPs representing about 1% of total coding genes through gene duplication and gene diversification events. Based on their phylogeny and homology, plant CYPs have been grouped into single and multiple family, clans and subfamilies (Nelson et al., 1996). The P450 superfamily in plants can be categorized into two distinct clades: A-type and non-A-type, which are typically classified into six single-family clans and four multiple-family clans (Nelson et al., 2006; Durst and Nelson, 1995). The *Gm*P450s superfamily is also grouped into two types: A-type and non-A-type. A-type *Gm*P450s contain a single clan (Clan 71) divided into 20 families. These 20 families contain 197 *Gm*P450s. Non-A-type of *Gm*P450s are divided into 9 clans (clan 51, clan 72, clan 74, clan 85, clan 86, clan 97, clan 710, clan 711, clan 727) which are further divided into 28 families. These 28 *Gm*P450 families contain 149 *Gm*P450s. In soybean, the CYP71 is the most extensive *Gm*P450s family, with 53 members, while the CYP82 family comprises 25 members, making it the second largest family (Khatri et al., 2022).

CYPs act as versatile catalysts regulating various physiological processes through a plethora of biosynthetic and detoxification reactions (Coon, 2005). Cytochrome P450 monooxygenases (P450) are oxidoreductases, which participate in the biosynthesis of secondary metabolites, antioxidants, and phytohormones via *N*-dealkylation, C–H bond hydroxylation, *N*-hydroxylation, *S*-oxidation, reduction, decarboxylation, dimerization, desaturation, epoxidation of numerous exogenous compounds, ring extensions and C–C cleavage (Guengerich and Munro, 2013; Pandian et al., 2020). CYPs are heme-thiolate harbouring enzymes having crucial roles in growth, development and plant defense via synthesis of primary and secondary metabolites, fat metabolism, phytohormone biosynthesis and homeostasis, antioxidant activity, and xenobiotic metabolism (Guengerich, 2001; Zhang and Li, 2017).

Throughout their life cycle, plants are exposed to many abiotic and biotic stressors that negatively impact their growth, development, and productivity. Among biotic stresses, pest infestation in plants results in a nutrient-deprived condition, a decline in photosynthetic rate and crop loss. To cope with intruders, plants have evolved with complex defense mechanisms consisting of constitutive and induced defense mechanisms (Karban et al., 1997: Thomma et al., 1998; Kessler and Baldwin, 2002). However, CYPs reinforce chemical and molecular defense arsenals controlling secondary metabolite’s biosynthesis, regulating biosynthesis and homeostasis of phytohormones and elevating reactive oxygen species (ROS) scavenging (Walling, 2000; Rojo et al., 2003).

The engagement of CYPs in the production of cyanogenic glucosides, cutin and lignin could have a direct or indirect connection with plant defense arsenals against various biological stressors (Pinot and Beisson, 2011). For example, *AtCYP82G1* is associated with the biogenesis of monoterpene volatiles and function as a defensive agent against herbivores (Lee et al., 2010). Also in poplar plants, *CYP79D7* and *CYP79D6* prompt the biosynthesis of feeding deterrents and toxins. Moreover, these enzymes induce the release of volatile compounds (aldoximes), which act in indirect defense by repelling herbivores and attracting their natural foes (Irmisch et al., 2013). In *Populus trichocarpa*, the herbivore-induced production of aldomixes, was also reported to be regulated by the *CYP79D* gene family (Irmisch et al., 2015).

Similarly, *Verticillium dahliae* induced *CYP706B1* produces *(+)-δ-cadinene-8-hydroxylase* in cotton, which catalyse the biosynthesis of gossypol, a herbivore-defensive chemical (Luo et al., 2001). Resin acid is used in insect defence mechanisms in conifers (Hamberger et al., 2011). *CYP720B4* of Sitka spruce participates in diterpene metabolism and causes resin acid production. Also, it has been discovered that the *PAD3* belonging to CYP family, is indulged in camalexin production, a toxic phytoalexin that regulates resistance against green peach aphid (*Myzus persicae*) in *Arabidopsis* (Prince et al., 2014). Cembratriene-ol (CBT-ol) gets metabolised into cembratriene-diol (CBT-diol) by an unidentified CYP hydroxylase in trichome glands of *N. tabacum* plants. However, suppression of this CYP raised the CBT-ol content and showed resistance to aphids (*Myzus nicotianae*) (Wang et al., 2001). Moreover, CYPs are involved in the biosynthesis of DIMBOA and DIBOA (benzoxazinoids), which critically defend plants against pests, pathogens and weeds (Butrón Gómez et al., 2010; Dick et al., 2012; Kato-Noguchi et al., 2000). These reports indicated the critical functions of CYPs in attaining defense against plant-insect pests. Thus, CYPs are the potential targets for enhancing plant defense by employing metabolic engineering.

Soybean (*Glycine max* (L.) Merr.) holds an important place in agriculture as it is the fourth-largest grown crop and dietary staple food in Asian countries (Badole and Bodhankar, 2012). Soybean, is an economical important leguminous plant with high nutritional and therapeutic values, which offers a high percentage of protein (38-42%), edible oil (18-22%), and carbohydrates (17-19%) apart from unsaturated FAs (fatty acids), antioxidants, minerals, fibres and vitamins (Chauhan et al., 2002). Soybean is a multipurpose legume used in human and animal food, fodder and industrial purposes; enhancing its demand (Soyastats, 2010). Soybean is known for cancer prevention, reducing cholesterol and for growth and development as it contains a good amount of isoflavones, calcium and vitamins (Carrão-Panizzi and Erhan, 2007). Soybean aids in the development of soil fertility as it fixes atmospheric nitrogen via root nodules and enriches the soil by drooping leaves on the ground during maturity (Patil et al., 2018). Soybean being nutrient-rich with luxuriant growth, attracts many insect pests (Brahman et al., 2018). However, among several constraints to its production, insect pests negatively impact soybean quality and productivity (Cui et al., 1997). Polyphagous *Spodoptera litura* (Fab.), commonly known as common cutworm is a crucial defoliator pest that causes significant soybean crop loss due to its gregarious feeding. *S. litura* has broadened its distribution in the tropical and temperate areas of the Asia-Pacific islands as it has huge reproductive and migratory ability and infests more than 120 crop plants (Maree et al., 1999; Baskar et al., 2012). *S. litura’*s neonate larvae scrap chlorophyll from leaves while grown-up ones feed voraciously on soybean leaves and pods by making holes/leaving veins only (Bughio et al., 1994). This pest remains active through July-October synchronize with the soybean reproductive period; cause a loss of 30-50% in soybean grain yield (Marwoto, 2008; Vasudev and Sohal, 2016; Sundar et al., 2018). Therefore, the development of insect-pest tolerant varieties of soybean is in high demand for enhancing soybean production.

A thorough examination is being conducted in the current study of the *S. litura-*induced P450 cytochrome monooxygenase gene family in *G. max* to deduce their role in the insect-defense mechanism. *In silico* as well as *in vitro* studies related to phylogenetic and evolutionary analysis, gene duplication events, identification of *cis*-acting elements, protein-protein interaction network analysis and differential expression in diverse types of organs and gene expression analysis upon *S. litura*-infestation, wounding and foliar spray of signalling compounds, three-dimensional homology modelling, identification of conserved motifs, docking with heme cofactor required for their catalytic activity and enzyme-substrate interactions, depicted their responsiveness to biotic stress and allowed us to understand the functional mechanism of their action. This study on scrutinizing the role of the P450 cytochrome monooxygenase gene family in plant defense against *S. litura*, will generate some testable hypothesis for future functional studies which could help in generating insect resistance variety through genome modification and molecular assisted breeding.

## 2 Material and methodology

### 2.1 Selection of candidate GmCYP450s in *G. max* genome

A study on transcriptome analysis of *G. max* by, Wang et al., 2014 revealed the induction of hundreds of genes and transcript diversity in response to *S. litura* feeding. Out of diverse induced transcripts, we have selected biotic stress-responsive differentially expressed CYP450 genes to analyze their role in *G. max- S. litura* interaction. Protein and nucleotide sequences of selected 16 *GmCYPs* were fetched from the Phytozome database version 13 (https://phytozome-next.jgi.doe.gov/) and also manually evaluated using NCBI-BLAST tool (https://blast.ncbi.nlm.nih.gov/Blast.cgi). Molecular weight (MW), isoelectric point (pI), instability index, and hydrophilicity (GRAVY value) of candidate sequences were computed employing the ProtParam tool (https://web.expasy.org/protparam/) of ExPASy (https://www.expasy.org/). ExPASy server UniProtKB/Swiss-Prot (https://www.expasy.org/resources/uniprotkb-swiss-prot) was used to calculate the length of amino acids for all the listed GmCYPs sequences (Gasteiger et al., 2005). ExPasy-Prosite server was used to identify functional sites. Deep-Loc Tool (https://services.healthtech.dtu.dk/service.php?DeepLoc-1.0) was employed to analyze the subcellular localization of listed GmCYPs to gain insight into their functional mechanisms. Subcellular localization of all GmCYPs was represented as a heatmap using TBtool software. Subsequently, sequences were uploaded on the TMHMM version 2.0 online tool (https://services.healthtech.dtu.dk/service.php?TMHMM-2.0) for trans-membrane domain analysis (Wang et al., 2022).

### 2.2 Analysis of phylogenetic tree and gene duplication

To evaluate the evolutionary connections among leguminous dicots, model plants, monocots, homologous protein sequences of listed *GmCYPs* for *Arabidopsis thaliana, Nicotiana attenuata, Medicago truncatula* and *Oryza sativa* were acquired from NCBI database using BLAST search (https://www.ncbi.nlm.nih.gov/guide/proteins/). Subsequently, the Clustal omega tool was used to align amino acid sequences (https://www.ebi.ac.uk/Tools/msa/clustalo/). The maximum-likelihood phylogenetic tree was produced based on the result of multiple sequence alignments using MEGA 7.0 (Molecular Evolutionary Genetic Analysis) software (https://megasoftware.net/) with bootstrap replication value 1000, P-distance mode and partial deletion (Kumar et al., 2016). Finally, the phylogenetic tree was embellished, implying iTOL (https://itol.embl.de/upload.cgi) tool (Letunic and Bork, 2007).

MCScanX software was employed to identify duplicated CYP gene pairs found in the genomes of *G. max, A. thaliana, M. truncatula, N. atteneuata and O. sativa* through their non-redundant pairing, thereby generating five pairs (*G. max/G. max, G. max/A. thaliana, G. max/M. truncatula, G. max/N. atteneuata and G. max/O. sativa*) (Wang et al., 2012). The software employed an alignment file (generated by executing the all-versus-all BLASTP program for each of five pairs with e-value 10-5) and genome annotation data (retrieved from RefSeq of NCBI database) as input to generate a collinearity file. Collinearity file, which includes collinear pairs found in CYP genes for each species pair, was utilised to map duplicate gene pairs through the Circos software (http://circos.ca/software/) package (Krzywinski et al., 2009). To investigate selection pressure employed in *GmCYPs* duplication, the Ka/Ks ratio (synonymous/non-synonymous substitution ratio) was calculated through PAL2NAL software (http://www.bork.embl.de/pal2nal/) (Suyama et al., 2006).

### 2.3 Gene structure, chromosomal mapping and conserved motifs analysis

Exon-intron organization of listed *GmCYPs* was illustrated using GSDS (Gene Structure Display Server) (http://gsds.gao-lab.org/), where genomic sequences of all the genes were aligned with their corresponding cDNA sequences. TBtool software was used to visualize the gene structure of 16 *GmCYPs* (Hu et al., 2015). Listed *GmCYPs* were mapped to soybean chromosomes based on the generic feature format version file (GFF) retrieved from the Phytozome database. Chromosomal position and relative distances of genes were spotted using Ritchie lab-Phenogram software (http://visualization.ritchielab.org/phenograms/plot) (Abbas et al., 2022).

Functional domain in *GmCYPs* was searched by submitting protein sequences to Pfam: Protein families database tool (http://pfam-legacy.xfam.org/). InterPro 91.0 database (https://www.ebi.ac.uk/interpro/) and NCBI Conserved domain database (https://www.ncbi.nlm.nih.gov/Structure/cdd/cdd.shtml) were also used for the detection of the conserved domains (Mistry et al., 2021). Later, MEME: Multiple Em for Motif Elicitation software (https://meme-suite.org/meme/doc/meme.html) was employed to predict conserved motifs and generate logo plots of listed *GmCYPs* sequences selecting the number of motifs: 10 and rest parameters as default (Bailey et al., 2009). Multiple sequence alignment (MSA) with amino acid sequences of candidate *GmCYPs* was performed to investigate existing conserved motifs utilizing Clustal Omega (https://www.ebi.ac.uk/Tools/msa/clustalo/).

### 2.4 Analysis of cis-acting regulatory elements analysis in gene promoter regions, Trascription factor binding sites and miRNA target analysis

The 1000 bp nucleotide sequences upstream of translational start codon of each listed *GmCYPs* were retrieved from Phytozome v13 database. The cis-acting elements in listed *GmCYPs* sequences were predicted employing the Plant Care database (http://bioinformatics.psb.ugent.be/webtools/plantcare/html/). Further, the results of cis-acting element was analysed using TBtool software (Zhang et al., 2022). psRNA Target tool (https://www.zhaolab.org/psRNATarget/; Lescot et al., 2002) was employed for anticipation of miRNA that may target and regulate *GmCYPs* expression with default parameter settings (Dai and Zhao, 2011). The mature sequences of miRNA of *G. max* were accessed from the miRBase i.e. miRNA database (https://mirbase.org/). The *GmCYPs* mRNA sequences and miRNA sequences were submitted to psRNA Target analysis server to characterize *GmCYP’s* miRNA target sites (Chen et al., 2020, Dai and Zhao, 2011).

Transcription factor (TF) binding sites prediction in the *GmCYPs* promoter region was executed using PlantTFDB online server (http://planttfdb.gao-lab.org/); using default parameters and *p-*value of ≤ 1e-6 (Malviya et al., 2023). 

### 2.5 Plant-organ-specific gene expression profiling and protein-protein interaction

To analyse the organ-specific expression profiles of genes, RNAseq data related to differential expression in different tissues was retrieved from SoyBase Expression Explorer for each *GmCYPs* (https://www.soybase.org/expression). Expression values were measured as Reads/Kb/Million (RPKM)) for various organs, including the seed, stem, nodule, flower, young leaf, and green pod (Waese et al., 2017). Normalized RPKM values of the listed *GmCYPs* were used to generate a heat map using TBtool.

The protein-protein interaction network of *GmCYPs* was deduced using the String database (https://string-db.org/). For PPI, *G. max* was selected as the model plant, with medium confidence = 0.400 and the remaining parameters at the default settings (Szklarczyk et al., 2021).

### 2.6 Expression analysis of *GmCYPs* upon *S. litura* infestation and wounding

#### 2.6.1 Plant growth and maintenance

*Glycine max* seeds (Pusa 9712) were procured from ICAR-Indian Institute of Soybean Research, Indore, India. Seeds were surface sterilized after O/N soaking and sown in plastic pots in a plant growth chamber using standard protocols, i.e. a photoperiod of 16/8h light/dark cycle, 28°C temperature, 55-60% humidity and watered regularly.

#### 2.6.2 *S. litura* rearing and sample preparation

*S. litura* pupa were procured from ICAR–NBAIR (National Bureau of Agricultural Insect Resources), India and reared under standard conditions at temperature: 28°C; relative humidity; 65-70% and 14/10h light/dark cycle. For *S. litura*-infested sample collection, the 4^th^ instar larvae were starved O/N and released on 30 days old healthy soybean plants (One larva per plant) for feeding, whereas plants not infested by larvae were treated as control. Mechanical wounding was accomplished as per Singh et al., 2008. After 24 h of infestation/wounding, the entire shoot of control and treated plants were harvested from 3 independent experiments and immediately frozen at -80°C for further experimentation.

#### 2.6.3 RNA extraction and quantitative qPCR

Trizol reagent (Sigma Aldrich, US) was utilized to extract total RNA from both control and treated samples following the manufacturer’s guideline and as per previously described protocols (Keshan et al., 2021; Singh et al., 2021b; Singh et al., 2018). RNA was quantified using UV-spectrophotometer (Biorad), and quality was analyzed on 2% agarose gel. The first-strand cDNA was constructed utilizing the cDNA synthesis kit, (Biorad) following the manufacturer’s instructions. Primer 3 (v.0.4.0)10 tool was employed for designing specific primers, and CFX Opus 96 Real-Time PCR (Biorad) was used for RT-PCR. Each RT-PCR reaction was conducted in SYBR green supermix with 10 mM solution of each gene-specific primer (0.5 mL), 1μL of diluted cDNA and 3 μl of RNase-free water. A soybean housekeeping gene, elongation factor 1(EF-1), was used as an internal reference gene (Saraiva et al., 2014).

### 2.7 Analysis of gene expression *GmCYPs* upon foliar spray with signalling compounds

Foliar application of an equal volume of 50 mM ethephon, 5 mM salicylic acid, 100 mM of methyl-jasmonate, and 0.5% (v/v) ethanol in water/only water was administrated on one-month-old healthy soybean plants kept in individual enclosures under identical conditions (Singh et al., 2008; Yadav et al., 2022). The treated and control samples were collected after 24 hours and immediately frozen at -80°C. The samples were collected in biological triplicates for subsequent analysis. Data for gene expression analysis was interpreted employing the 2^-ΔΔCT^ method. T-test (P < 0.05) and ANOVA were applied for statistical analysis, while differentially expressed genes were selected based on a criterion of more than two-fold, either induction or reduction.

### 2.8 Homology modelling of selected *GmCYPs* and docking with heme ligand

Three-dimensional structures of *GmCYPs* were predicted using Phyre2, Swiss modeller and i- TASSER by submitting protein sequences. Structures were analyzed based on different parameters such as query coverage, template size and origin, stability of the predicted proteins and validated by Ramachandran plot (Kelley et al., 2015; Waterhouse et al., 2018; Zhang, 2008). After which, the best model were utilized for further analysis such as identification of conserved motifs and highlighting them on the most accurately modelled structures using Pymol (DeLano, 2002). Docking of heme prosthetic factor with one of the randomly selected GmCYP was executed using Maestro Glide docking software (Schrodinger’s tool) (Bhachoo and Beuming, 2017). Prior to docking, ligand (Heme) and GmCYP structures were optimized by Maestro’s Ligprep and Protein Preparation wizard, respectively. Receptor grid generation was constructed by selecting residues of heme-binding motifs. *Arabidopsis* CYP94C1 was taken as a reference for this analysis.

### 2.9 Interaction of selected *GmCYPs* with their substrate

Information about substrates of *GmCYPs* was retrieved from literature and databases survey, and 3-D structures of *GmCYPs* substrates were fetched from PubChem. To show the interaction between *GmCYPs* and their substrates, the InChl format of the substrates fetched from PubChem was converted to. pdb format employing Open Babel-Chemical file format (http://www.cheminfo.org/Chemistry/Cheminformatics/FormatConverter/index.html) (O’Boyle et al., 2011; Kim et al., 2019). Patch Dock was applied to perform interaction studies with parameters: RMSD value= 4 and protein-small ligand complex type, and 100 top results. The ten highest-rank Patch dock docking outcomes were refined through FireDock (Mashiach et al., 2008; Schneidman-Duhovny et al., 2005).

## 3 Results

### 3.1 Sequence retrieval and *in silico* characterization of *GmCYP450s*

Gene IDs for all S. litura-induced *GmCYPs* were accessible from NCBI and phytozome database, from where their protein and nucleotide sequences were retrieved. A self-BLAST search was also conducted to eliminate redundant sequences that finally identified 16 of the *S. litura*-inducible *GmCYP450*s involved in biotic stress response. The naming of genes was based on their homology to *Arabidopsis CYP450s* and Phytozome gene description. Detailed information of 16 candidate *GmCYPs*, including gene ID, Phytozome ID, Uniprot ID, and the number of peptide sequences along with physiochemical properties, are represented in **Table 1**. The candidate *GmCYPs* range from 6152 bp (*GmCYP78A5*) to 2164 bp (*GmCYP78A3*), and CDS sequences ranged between 1398 bp (*GmCYP85A1*) to 1746 bp (*GmCYP78A5*). The full length of peptide sequences varied from 466 (GmCYP85A1) to 582 (GmCYP78A5) amino peptides with molecular weight (MW) varying from 53.375 (*GmCYP85A1*) to 65.343 (*GmCYP78A5*) kDa and their isoelectric point (pI) values varying from 6.54 (*GmCYP82A2*) to 9.27 (*GmCYP90A1*), indicating that maximum proteins were basic. However, instability index values indicate that most *GmCYPs* are stable (Instability index > 40) except *GmCYP71AU50*, *GmCYP93A3*, *GmCYP89A2*, and *GmCYP71D10*. In addition, 13 genes were hydrophilic (GRAVY value less than zero), while *GmCYP78A5-like*, *GmCYP76C1-like* Determination of subcellular localization were predicted using reticulum (ER**).** Their presence was also predicted in the chloroplast, mitochondria, cell membrane, vacuole, golgi apparatus and nucleus with minimal expression, as depicted in **Supplementary Figure 1**.

**Table 1:** List of 16 *GmCYPs* and their physiochemical properties.

Gene Name	Phytozome ID	Uniprot ID	Peptide sequences	Molecular weight (kDa)	pI	Instability index	Aliphatic Index	GRAVY
GmCYP98A2	Glyma.19G126000.1	O48922	510	57.674	8.58	36.45, Stable	93.28	-0.19
GmCYP82A2	Glyma.13G285300.1	O81972	523	58.715	6.54	36.18, Stable	100.06	-0.059
GmCYP78A4-like	Glyma.02G119600.1	I1JEG7	517	58.316	6.96	35.84, Stable	94.4	-0.002
GmCYP98A2-like	Glyma.03G122000.1	I1JN04	510	57.706	8.72	36.28, Stable	92.32	-0.198
GmCYP71A1	Glyma.05G042600.1	I1K057	513	58.483	9.01	36.11, Stable	91.58	-0.108
GmCYP78A5	Glyma.06G310800.1	K7KYE8	582	65.343	8.06	38.15, Stable	86.95	-0.129
GmCYP78A3	Glyma.07G052300.1	I1KHS1	543	61.033	8.9	33.48, Stable	89.24	-0.06
GmCYP78A5-like	Glyma.08G104100.1	I1KS17	515	57.914	9.01	39.69, Stable	96.75	0.021
GmCYP71AU50	Glyma.08G140500.1	I1KT85	501	57.199	8.69	42.93, Unstable	95.92	-0.204
GmCYP93A3	Glyma.10G092500.1	A0A0R0HYP7	510	57.967	6.67	42.3, Unstable	94.62	-0.187
GmCYP76C1-like	Glyma.11G108300.1	I1LJ26	508	54.135	7.14	32.24, Stable	102.11	0.030
GmCYP90A1	Glyma.11G228900.1	K7LRQ4	473	53.942	9.27	39.8, Stable	93.18	-0.137
GmCYP78A6-like	Glyma.16G021200.1	I1MKF4	537	60.415	7.66	32.05, Stable	93	0.038
GmCYP85A1	Glyma.19G033900.1	I1N6E7	466	53.375	9	34.85, Stable	87.83	-0.185
GmCYP89A2	Glyma.20G018800.1	A0A0R4J634	513	59.257	8.59	45.82, Unstable	97.79	-0.13
GmCYP71D10	Glyma.15G050300.1	O48923	511	58.462	8.59	45.98, Unstable	92.67	-0.222

### 3.2 Analysis of phylogenetic and gene duplication events

**Figure 1:**
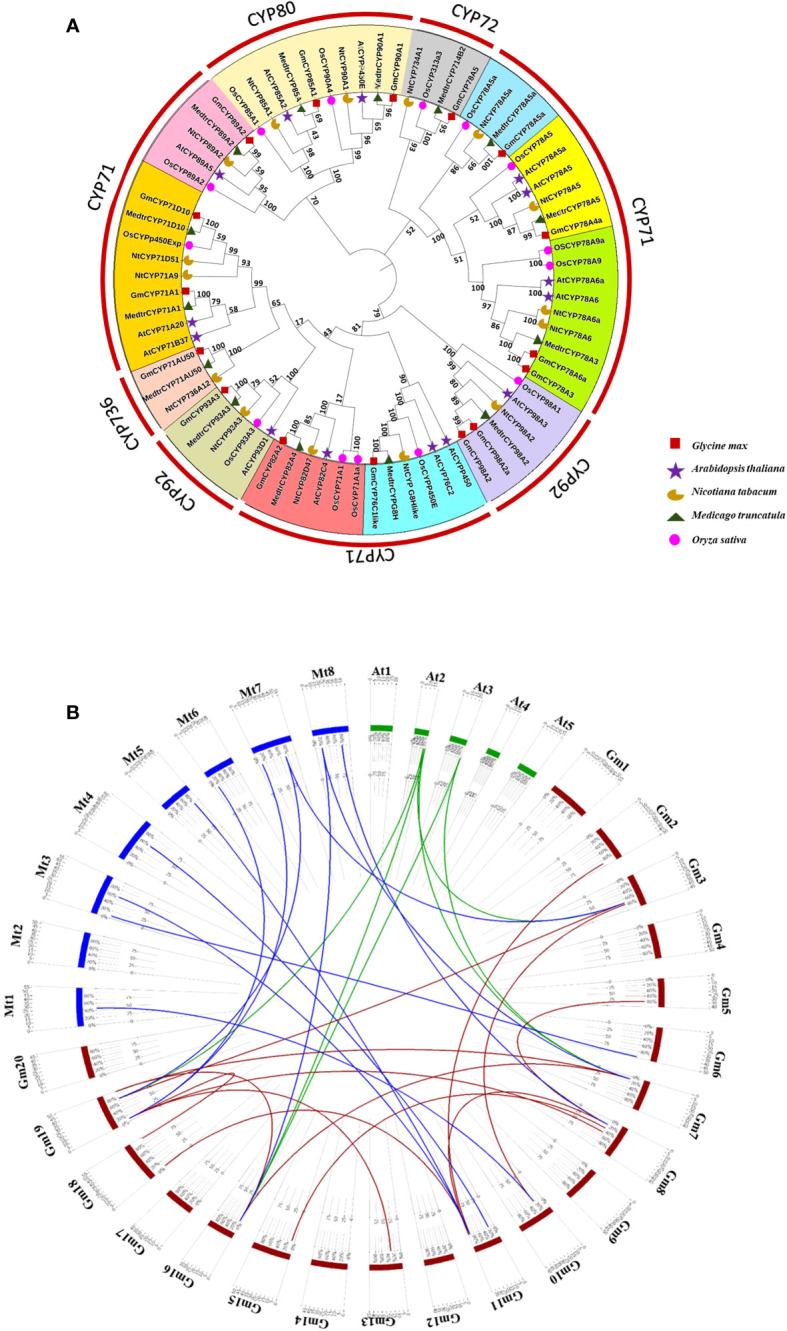
**(A)** Phylogenetic analysis of selected CYP450s of *Glycine max, Arabidopsis thaliana, Nicotiana tabacum, Oryza sativa and Medicago truncatula.* Full length protein sequences were aligned using Clustal omega and the tree was constructed by maximum likehood method using MEGA-X with 1000 bootstrap replication value. Different groups (representing different families) were coloured. *Glycine max* CYP450s (*GmCYPs*), *Medicago truncatula* CYP450s (MedtrCYPs), *Nicotiana tabacum* CYPs (NtCYPs), *Oryza sativa* CYPs (OsCYPs), and *Arabidopsis thaliana* CYPs (AtCYPs) are represented with brown square, green triangle, yellow half moon, red circle and blue star respectively. Bootstrap values are also represented. **(B)** Circos showing duplicated CYPs gene pairs between *G. max* and *A. thaliana* (green), *G. max* and *M. truncatula* (blue) and for *G. max* (brown).

A phylogenetic tree was constructed from *G. max* CYP450s genes (16) and their homologous members from *A. thaliana* (14)*, M. truncatula* (14), *O. sativa* (14) and *N. tabacum* (15); to understand the evolutionary relationship among them (**Figure 1A**). The findings show that CYPs have evolved dramatically throughout the time series, as seen by distinct clades in tree, implying that CYPs play a variety of functions in various plant systems. These proteins were categorized into 12 clades; among them, Clan 71 has a maximum of 45 members, and Clan 80, Clan 72, Clan 92, and CYP736 contained 10, 4, 11 and 3 members, respectively. *GmCYPs* were dispersed across the phylogenetic tree and belonged to different clades illustrating evolutionary relatedness with CYPs from *A. thaliana, M. truncatula*, *O. sativa* and *N. tabacum.* However, the maximum like-hood tree indicated that *G. max* and *M. truncatula* are closely related evolutionarily (having the highest bootstrap values), inferring they are members of the same family. Gene duplications are crucial for expanding and evolving gene families, driving species divergence and new gene functions (Innan and Kondrashov, 2010). Examination of gene duplication occurrence in the *G. max* genome revealed that 15 pairs of *GmCYPs* underwent segmental duplication i.e. duplicated genes were found on different chromosomes. A total of 6 pairs of duplicated genes between *GmCYPs* and genes from *A. thaliana* while 12 pairs of duplicated genes between *GmCYPs* and genes from *M. truncatula* were observed (**Figure 1B**). The selection pressure was interpreted by calculating Ka/Ks which varied from 0.04918 (NP_850337.1/XP_003521103.1) to 0.332959 (XP_006582383.1/XP_039688169.1) (**Supplementary Table 1**).

### 3.3 Gene structure, chromosomal localization and motif analysis

To comprehend the structure of putative *GmCYPs*, their intron-exon organization was predicted, which revealed that *GmCYPs* gene structures were diverse, with the exon number varying from 1 to 9. *GmCYP89A2* was predicted to be intron-less (single exon) whereas *GmCYP85A1*, *GmCYP90A1* and *GmCYP78A5* consists of 8, 7, 6 introns and 9, 8, 7 exons respectively. *GmCYP93A3*, *GmCYP93A2* and *GmCYP93A2-like* genes contained 3 exons and 2 introns, while the rest of them have 2 exons with a single intron (**Supplementary Figure 2**). This gene structure analysis helps to determine evolutionary, structural and functional characteristics of genes. Chromosomal mapping of *GmCYPs* illustrated that these *GmCYPs* were randomly placed on 13 (out of 20) soybean chromosomes. Chromosomes 8, 9 and 11 contained 2 *GmCYPs*, whereas the remaining 10 chromosomes contained a single gene. *GmCYPs* are located on chromosome arms except for *GmCYP98A2*, *GmCYP98A2-like* and *GmCYP93* implying their frequent participation in recombination process (**Supplementary Figure 3**, **Supplementary Table 2**).

Conserved motif and domain analysis is a useful method for protein function prediction (Watson et al., 2005). All the listed *GmCYPs* were examined and analyzed with Pfam, NCBI-CDD and Inter-pro databases for the presence of functional domains; investigated the existence of the P450 domain (conserved in cytochrome family with CDD and Pfam: PF00067, Inter pro: PS00086 id). SMART and TMHMM executed the presence of trans-membrane regions in all listed *GmCYPs*. Additionally, all genes also harboured Cytochrome P450 Cysteine heme iron ligand signature: IPR001128, detected by ExPasy-Prosite server. In our study, MEME online tool detected the presence of 10 possible motifs (**Supplementary Figure 4**). Motif 1, 2, 3 and 4 appeared in all candidate *GmCYPs* representing conserved motifs of the P450 superfamily: heme-binding region (FXXGXRXCXG), PXRX motif, I-helix oxygen-binding domain (AGxDT) and K-helix region (EXXR). Exploration of listed *GmCYPs* for conserved motifs was executed by MSA and identified these four conserved motifs (**Supplementary Figure 5**).

### 3.4 Cis-acting element, TF binding site and miRNA target analysis

**Figure 2:**
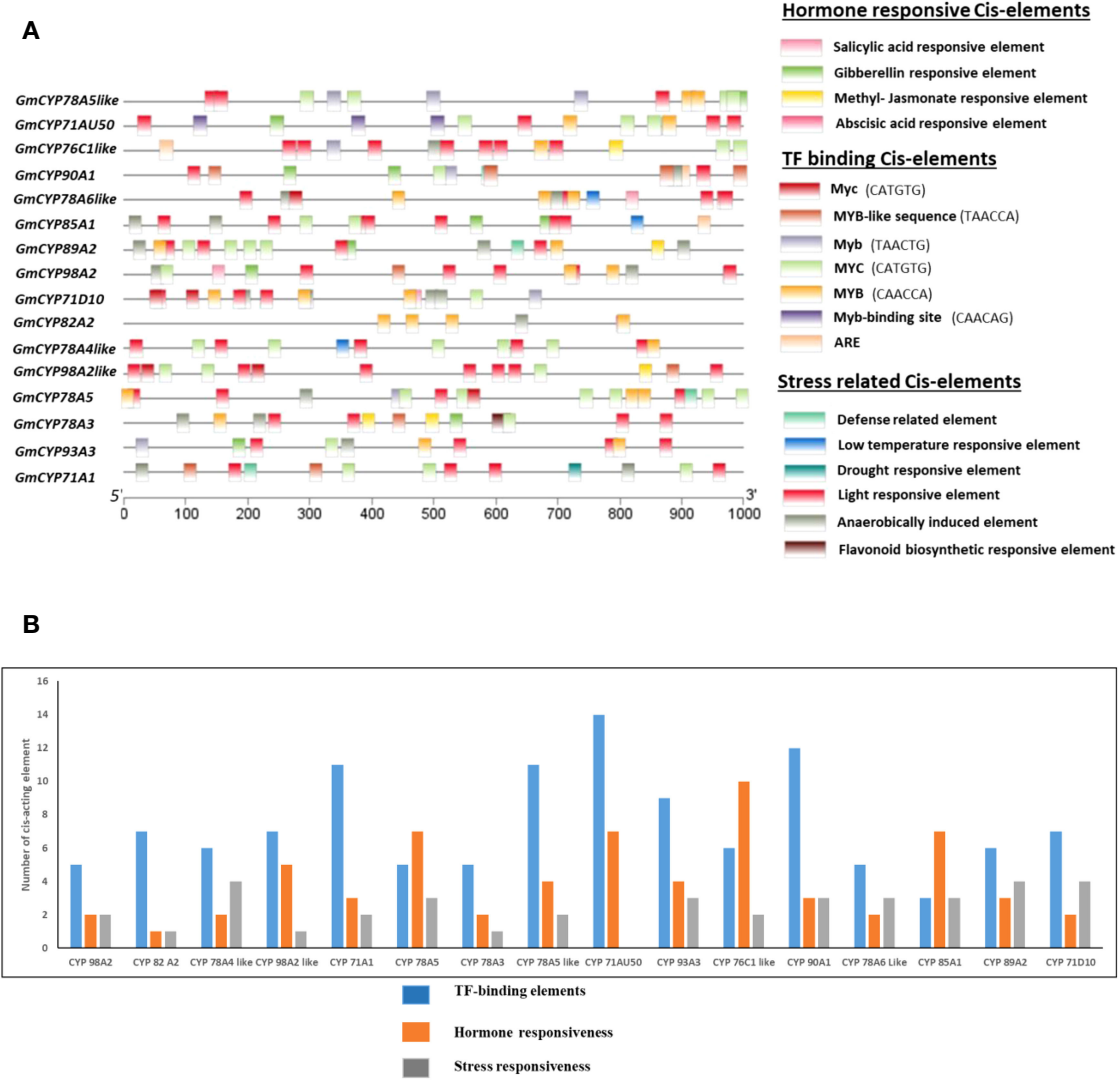
**(A)** Identification of Cis-acting elements in 16 *GmCYPs* promoters. Predicted Cis-elements of *GmCYPs* were depicted in different coloured boxes which are categorized into three categories: Hormone responsive, stress related and TF factor binding. Relative position of cis-acting elements based on start codon (ATG) can be estimated using scale. **(B)** and graph is showing number of cis-element involved in TF-binding elements, hormone responsiveness and stress responsiveness for each 16 *GmCYPs*.

Cis-acting elements present in the promoter region may be crucial in controlling the transcription of genes in response to environmental stressors and plant growth. A comprehensive analysis was executed to decipher cis-element analysis existing in the promoter region of 16 *GmCYPs*. Several cis-acting elements, including those responsive to stress, hormones and TF binding, were identified in the promoter regions of *GmCYPs*, where each *GmCYPs* harbours more than one cis-acting element (**Figure 2**). Analysis revealed that these 16 *GmCYPs* harbour 88 light-responsive elements, which mainly include Sp1, sbp-CMA1c, TCT-motif, TATA-box, GATA-motif, I-box, Box 4, Gap-box, MRE, G-box, AE-box, chs-CMA2a, chs-CMA1a, GATA-motif, Sp1, GT1-motif, ATC-motif, AE-box, ATCT-motif, 3-AF1 binding site, ABRE3a, CACGTC, TCTTAC, TTACTTAA, GATAGGA motif. However, 46 phytohormone-responsive cis-elements were predicted; 16 methyl jasmonate hormone-responsive elements (TGACG-motif and CGTCA-motif), 14 ABA-responsive elements (ABRE), 10 Gibberellin hormone-responsive elements (TATC-box, CCTTTTG, P-box, GARE-motif), 3 Auxin-responsive elements **(**AuxRR-core and TGA-element), and 3 Salicylic acid-responsive elements (TCA-element). The presence of 3 low temperature (LTR), 22 Anaerobic conditions- responsive (ARE), 5 defense- responsive (TC-rich repeats), 5 drought-responsive elements and 1 flavonoid biosynthesis regulating (MBSI) cis-acting elements were detected. Additionally, binding sites of TFs on the *GmCYPs* promoter region were uncovered using PlantTFDB tool. Here, 14 types of transcription families were predicted in 11 *GmCYPs* promoter regions (**Supplementary Figure 6**) while *GmCYP90A1*, *GmCYP89A2*, *GmCYP78A6-like* and *Gm78A4-like* were deprived of any TF binding sites. AP2 family were predicted in *GmCYP76C1-like, GmCYP78A5-like and GmCYP98A2-like*; ERF was found on *GmCYP76C1-like* and *GmCYP82A2*; Bzip was restricted to *GmCYP76C1-like*; NAC in *GmCYP93A3, GmCYP76C1-like* and Dof in *GmCYP76C1-like*, *GmCYP93A3, GmCYP78A5-like* and *GmCYP85A1.*

miRNA in eukaryotes is a non-coding, short RNA comprising 21-24 nucleotides that bind to and cleaves mRNA for post-transcriptional regulation (Jones-Rhoades et al., 2006). Using *G. max* miRNA library, miRNA target analysis for *GmCYPs* was performed using the psRNATarget tool; it revealed 16 *GmCYPs* as putative targets of 94 miRNAs when E= 5.0 (**Supplementary Table 3**). *GmCYP78A5* was targeted by the largest numbers of miRNA, 23 followed by *CYP90A1* (11 miRNA), *CYP93A3* (9 miRNA). In this analysis, we can conclude that miRNAs possessed diversity in regulating *GmCYPs*, where miRNAs inhibit *GmCYPs* mainly through cleavage and some via translation.

### 3.5 Organ-specific gene expression profiling

**Figure 3:**
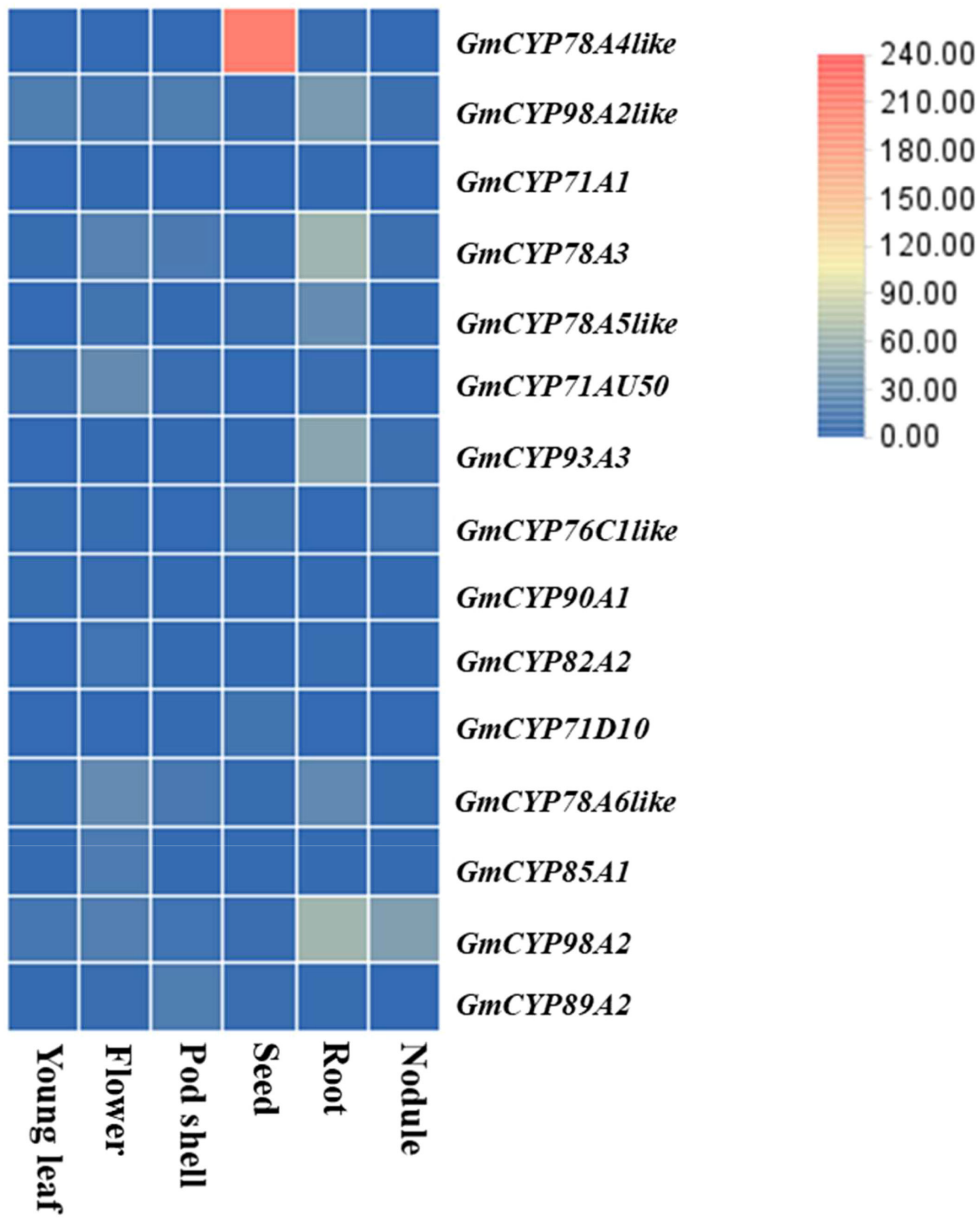
Organ-specific Expression profile of *GmCYPs* of *Glycine max* is represented in the form of heat map. Normalized RPKM values of *GmCYPs* were retrieved from Soybase Expression Explorer. (Expression data was not available).

To gain insight into regulatory functions of genes controlled by a specific plant-organ, RNA-seq expression data (RKPM Values) of listed *GmCYPs* were procured from soybean expression explorer for investigating *GmCYPs* expression into six different plant-organ i.e. seed, young leaf, nodule, root, flower, pod shell, seed (**Figure 3**). The results exhibited that all the 15 *GmCYPs* were expressed at least in one plant-organ, and *GmCYP98A2*, *GmCYP98A2-like*, *GmCYP78A3*, *GmCYP78A6-like* showed high expression in all six plant-organs indicating their predicted roles in soybean growth, development and countering various environmental stresses. Furthermore, some *GmCYPs* showed organ-specific expression patterns, such as *CYP78A5* and *CYP93A3*, which exhibited augmented expression within the root, implying their role in the development of roots, interaction with plant microbes and stress tolerance. Similarly, *CYP71AU50* and *CYP85A1* were highly expressed in flowers, while *CYP89A2* showed substantially greater expression in pod shells, implying their functions in reproduction/floral-related stimuli. Additionally, *CYP71D10* and *CYP76C1-like* were found to be explicitly expressed in seeds, indicating their role in seed development and maturation. On the other hand, *CYP78A4-like* was expressed only in two plant-organs, seed and root, with a higher level of expression in the seed. Finally, *CYP71A1* showed minimal expression in the flower and root, suggesting its possible involvement in other plant organs or stages of soybean development. However, it should be noted that expression data of *GmCYP78A5* was unavailable, which limits our understanding of its organ-specific expression pattern.

### 3.6 Protein network, enrichment analysis and GO annotation

**Figure 4:**
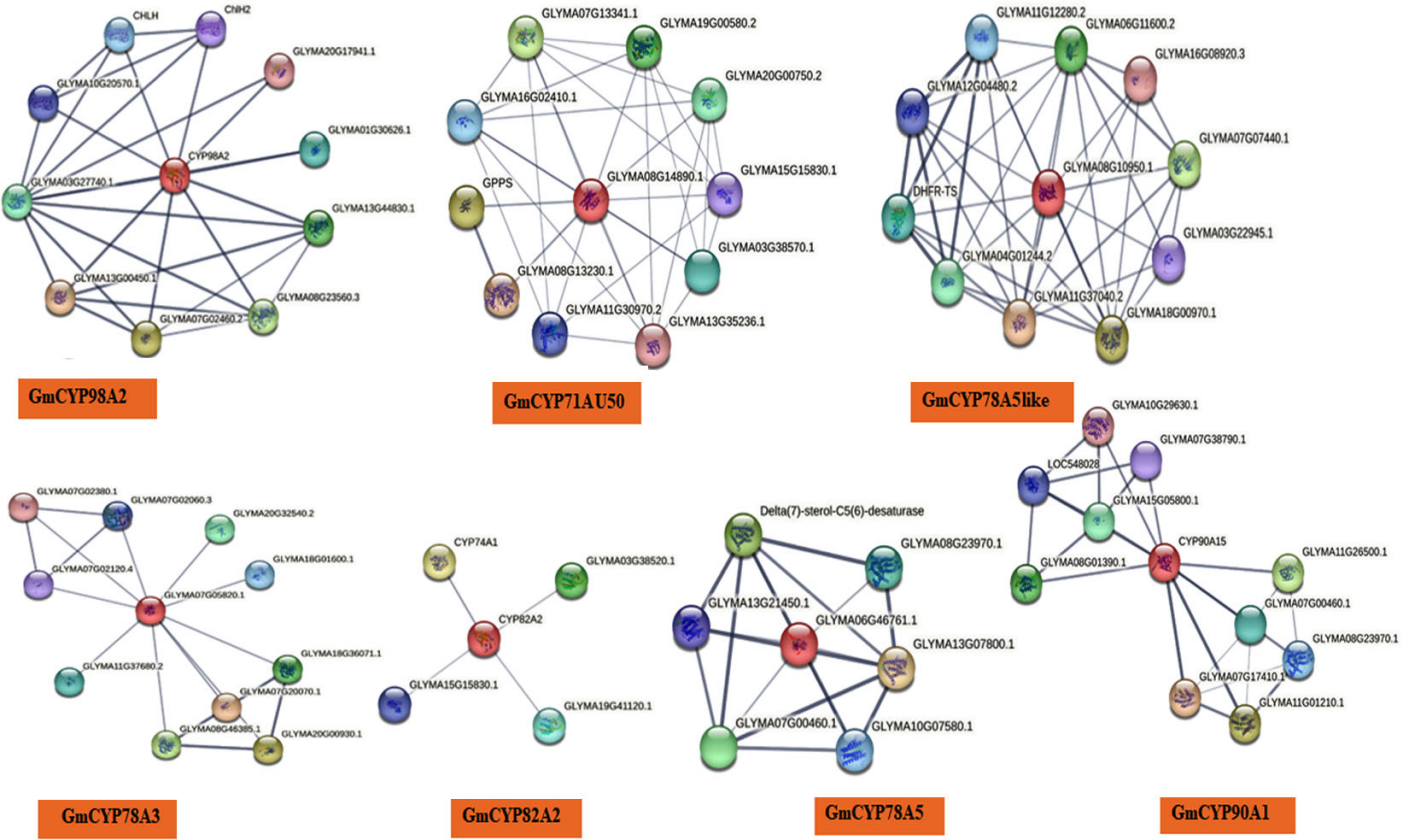
Protein-protein interactions (PPI) network of *GmCYPs* (which showing interaction with other stress responsive proteins). String database was utilized for this analysis.

Protein-protein interaction (PPI) analysis of each *GmCYPs* with other *G. max* proteins was predicted using the STRING database. PPI analysis is a comprehensive method of knowing predicted protein function. Out of 16 *GmCYPs*, only 8 *GmCYPs* have shown interactions with other *G. max* proteins involved in plant stress response (**Figure 4**). GmCYP98A2 exhibited interaction with GLYMA07G02460.2 (*Shikimate o-hydroxycinnamoyltransferase isoform x3*) GLYMA08G23560.3 (*Shikimate o-hydroxycinnamoyltransferase*) and GLYMA03G27740.1 (*5-O-(4-coumaroyl)-D-quinate 3’-monooxygenase*); involved in phenylpropanoid pathway. GmCYP82A2 showed its association with CYP74A1 (*Allene oxide synthase)*, which initiates the biosynthesis of jasmonic acid from hydroperoxides of free fatty acids derived from lipoxygenase, representing the first step in the process (Farmer and Goossens, 2019). GLYMA15G15830.1 (probable histone-arginine Methyltransferase 1), a positive regulator of oxidative stress tolerance, was predicted to interact with GmCYP82A2. STRING predicted GmCYP78A3 interaction with GLYMA07G02380.1 (AP2-like ethylene-responsive transcription factor), GLYMA07G20070.1 and GLYMA20G00930.1 (Checkpoint serine/threonine-protein kinase). GmCYP71AU50 exhibited interaction with GLYMA08G13230.1 (*Pathogen-inducible salicylic acid glucosyltransferase)*, GLYMA16G02410.1 (*CYP b5-like steroid binding domain-containing protein)*, and GPPS (*Geranylgeranyl diphosphate synthase, type ii)* (Barja and Rodriguez-Conception, 2021). GGPP is responsible for the biosynthesis of chlorophylls, carotenoids, or plastoquinones, which is vital for photosynthesis.

GmCYP90A1 exhibited interaction with brassinoide synthesizing enzymes; GLYMA11G07780.1 and GLYMA02G05780.2 (*3-epi-6-deoxocathasterone 23-monooxygenase)*, GLYMA07G17410.1 (*Steroid 5-alpha-reductase det2)*, GLYMA06G24540.1 *(*member of CYP450 family*).* It also interacts with GLYMA07G04470.1 (Typhasterol/6-deoxotyphasterol 2alpha-hydroxylase; belongs to the cytochrome P450 family) responsible for tyrosine and secondary metabolite synthesis. CYP78A5 interact with GLYMA13G07800.1 Delta(7)-sterol-C5(6)-desaturase isoform X1 and Shikimate o-hydroxycinnamoyl transferase involved in the biosynthesis of sitosterol and campesterol (Hoffmann et al., 2004). CYP78A5-like interacts with GLYMA11G37040.2 (Alpha-ketoglutarate-dependent dioxygenase alkb), GLYMA18G00970.1 (Alpha-ketoglutarate-dependant dioxygenase alkb, U) engaged in various reactions in plant metabolism such as flavonoid biosynthesis and ethylene biosynthesis (Zhang et al., 2004; Cheng et al., 2014). GLYMA16G08920.3 (heavy metal-associated isoprenylated plant protein 4) is involved in heavy metal detoxification and homeostasis mechanism. CYP85A1 interact with steroid 5-alpha-reductase (GLYMA07G17410.1 and GLYMA11G01210.1) involved in steroid biosynthesis and brassinolide biosynthesis (GLYMA02G05780.2 and GLYMA07G04470.1) and GLYMA11G26500.1 (long-chain fatty acid omega-monooxygenase), which is involved in the biosynthesis of complex polymer, sporopollenin.

### 3.7 Gene expression analysis of *GmCYPs* during *S. litura*-infestation and mechanical damage

**Figure 5:**
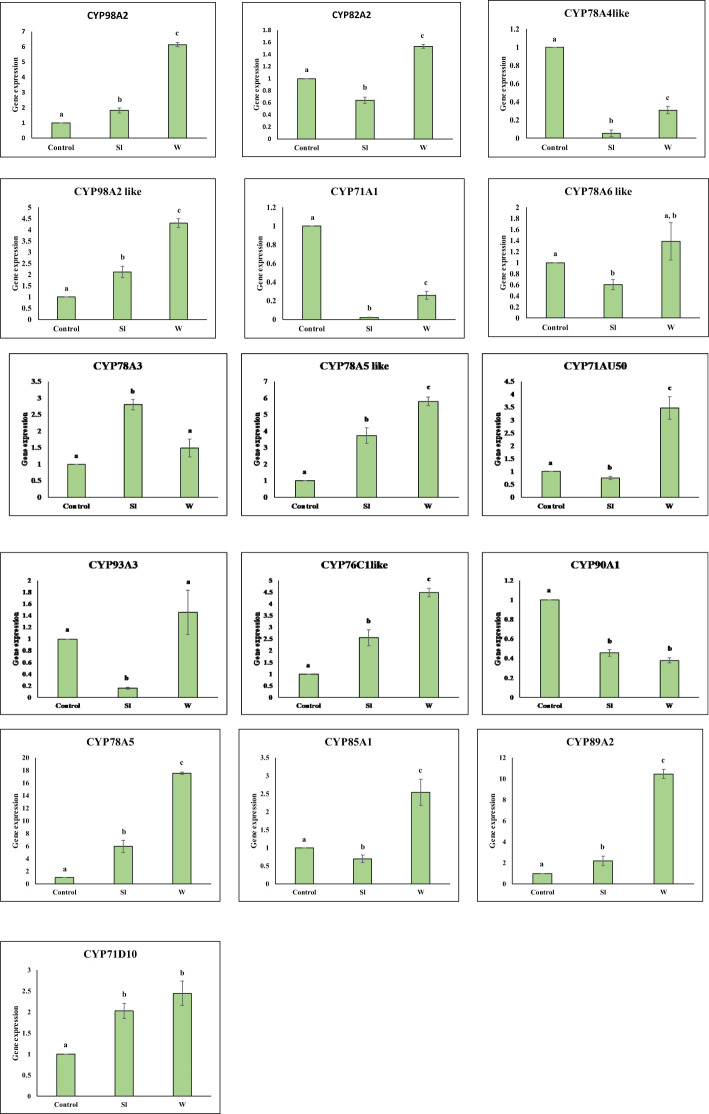
Graph showing expression profile of 16 *GmCYPs* upon Sl (*S. litura-*infestation) and W(wounding). The qPCR data were analyzed utilizing 2^-ΔΔCT^ method and statistical analysis was performed using ANOVA and Tukey’s test (P < 0.05). Bars were showing mean value whereas standard deviation were represented using error bars. a and b represents if two values are significantly different or not.

Gene expression analysis of 16 *GmCYPs* was executed by qPCR using gene-specific primers, the results indicated that 7 of *GmCYPs* were upregulated while 4 of the *GmCYPs* were downregulated on *S. litura* infestation. *GmCYP78A5* exhibited a maximum change in expression while *Gm78A5-like*, *Gm98A2-like*, *GmCYP78A3*, *GmCYP76C1-like*, GmCYP89A2 and *GmCYP71D10* showed upregulation whereas *GmCYP78A4-like*, *GmCYP71A1*, *GmCYP93A3* and *GmCYP90A1* showed downregulation during infestation. However, 9 out of 16 *GmCYP*s were upregulated on mechanical wounding, and 3 *GmCYP*s were downregulated. *GmCYP78A5*, *GmCYP89A2* and *GmCYP78A5-like* showed high gene expression on mechanical wounding (**Figure 5**).

### 3.8 Gene expression analysis of *GmCYPs* upon the foliar spray of signalling compounds

**Figure 6:**
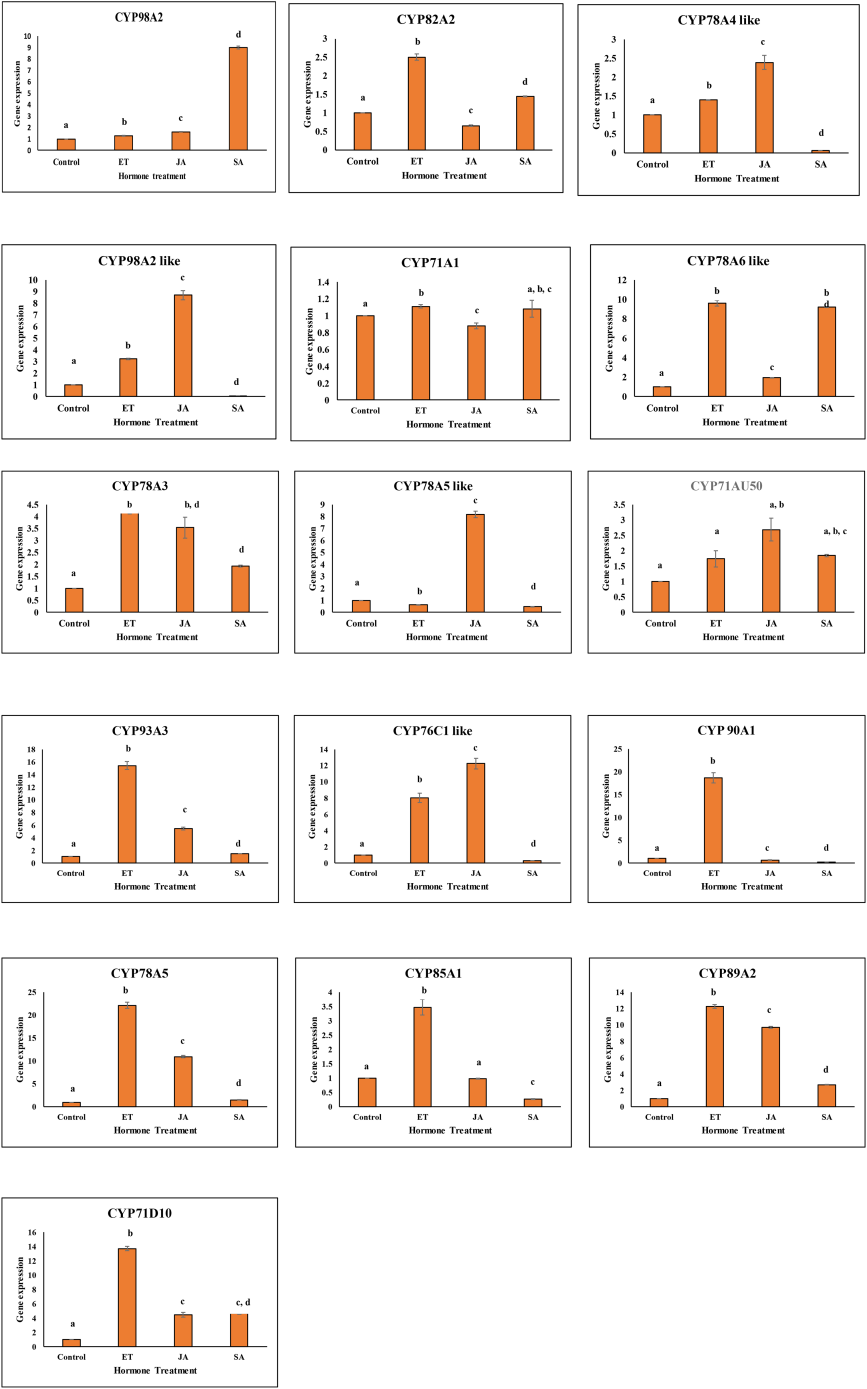
Graph showing expression profile of 16 *GmCYPs* on application of JA (Methyl-jasmonate), SA (Salicylic acid), and ET (Ethylene). The qPCR data were analyzed utilizing the 2 ΔΔ CT method and statistical analysis was performed using ANOVA and Tukey’s test (P < 0.05). Bars were showing mean values whereas standard deviation were represented using error bars.

Gene expression analysis of 16 *GmCYPs* was performed upon the foliar spray of ethylene, salicylic acid and methyl-jasmonate to acquire insights into the role of signalling compounds in regulating the expression of *GmCYP*s in plant defense. On ethylene treatment, 11 *GmCYPs* (*GmCYP82A2, GmCYP98A2-like, GmCYP78A5, GmCYP78A3, GmCYP93A3, GmCYP76C1-like, GmCYP90A1, GmCYP78A6-like, GmCYP85A1, GmCYP89A2, and GmCYP71D10*) whereas 5 *GmCYPs* (*GmCYP98A2, GmCYP78A4-like, GmCYP71A1, GmCYP78A5-like and GmCYP71AU50*) were upregulated and downregulated respectively. Similarly, methyl-jasmonate application induced expression of 10 *GmCYPs* (*GmCYP78A5-like, GmCYP98A2-like, GmCYP78A5, GmCYP78A3, GmCYP78A5-like, GmCYP71AU50, GmCYP93A3, GmCYP76C1-like, GmCYP89A2, and GmCYP71D10*). However, on SA application, 4 *GmCYPs* (*GmCYP71D10, GmCYP89A2, GmCYP78A6-like* and *GmCYP98A2*) were induced, while 6 *GmCYPs* (*GmCYP78A4-like, GmCYP98A2-like, GmCYP78A5-like, GmCYP76C1-like, GmCYP90A1 and GmCYP85A1*) were repressed, designating the involvement of SA directly or indirectly in gene regulation. (**Figure 6**).

Methyl-jasmonate foliar application upregulated four of the *S. litura* -induced *GmCYP*s, illustrating jasmonates’ role in regulating the *GmCYP*s expression upon *S. litura* -infestation. The presence of methyl-jasmonate sensitive *cis*-acting regulatory elements in the promoter region in these genes supported the notion that jasmonate is responsible for their regulation. Four *GmCYPs*, which had their expression elevated by ethylene treatment, were also induced by jasmonate. Jasmonate and ethylene are reported to act synergistically (Kessler and Baldwin, 2002).

### 3.9 Homology modelling of 16 *GmCYPs*

**Figure 7:**
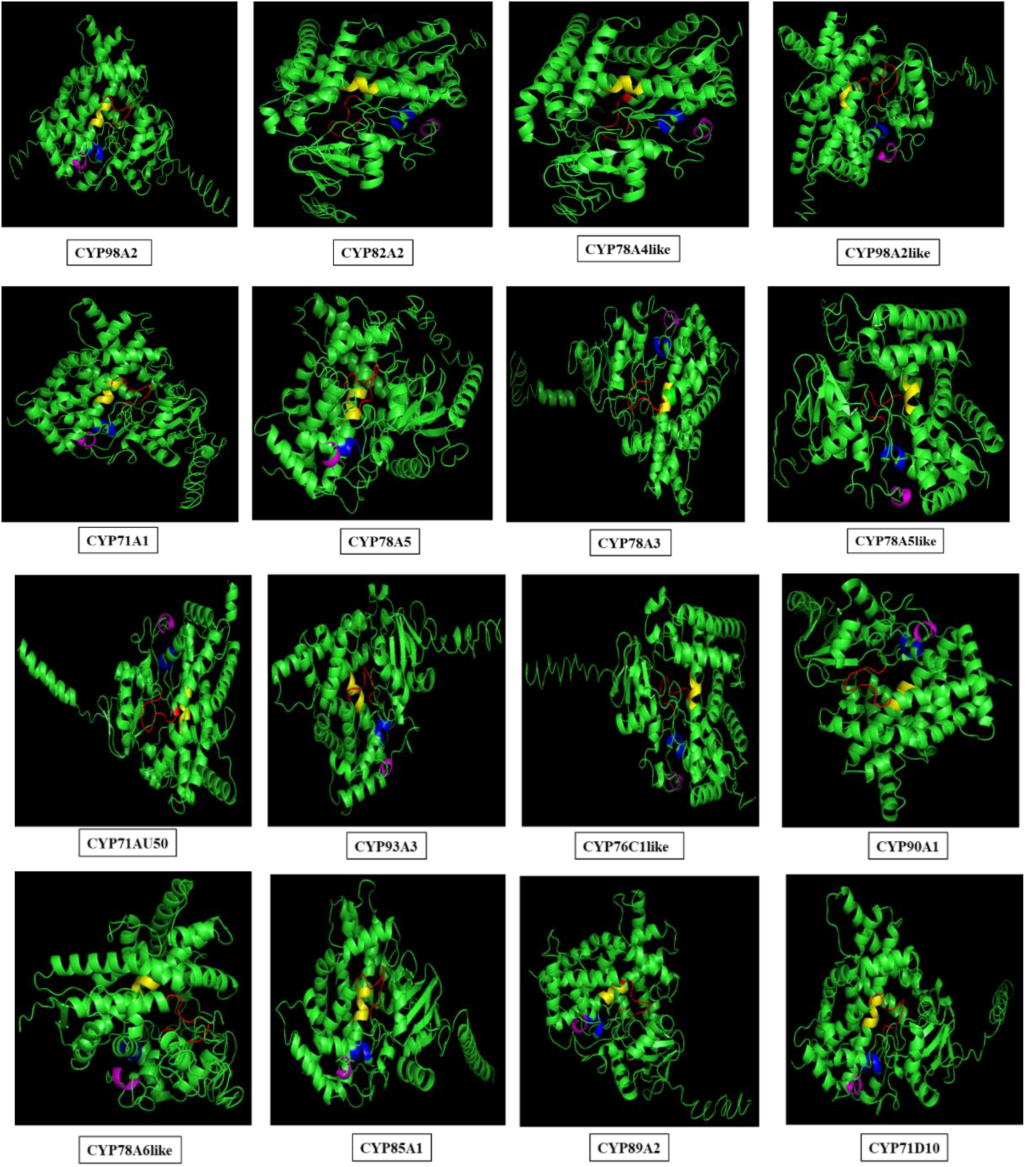
Three dimensional structures of selected 16 proteins. The structures are modelled using i-tasser server and conserved motifs were shown highlighted in red (FxxGxRxCxG), blue (ExxR), magenta (PxRW) and yellow (AGxDTT) color.

Three-dimensional (3-D) structures of differentially regulated *S. litura*-infested 16 *GmCYPs* were modelled using i-TASSER, and structures were corroborated by ensuring that >90% of the protein region fell within the permitted area of the Ramachandran plot. The template, coverage (>85%), Z-score, and estimated Tm; all these parameters are listed in **Supplementary Table 4**. All 16 *GmCYPs* chosen for analysis revealed the most typical arrangement of α-helix and β-sheets. The evolutionary conserved heme-binding region, PXRX motif, I-helix oxygen-binding domain and K-helix motifs of CYP450 proteins were highlighted using distinct colours (**Figure 7**).

### 3.10 Interaction of selected *GmCYPs* with their substrate and docking with heme ligand

**Figure 8:**
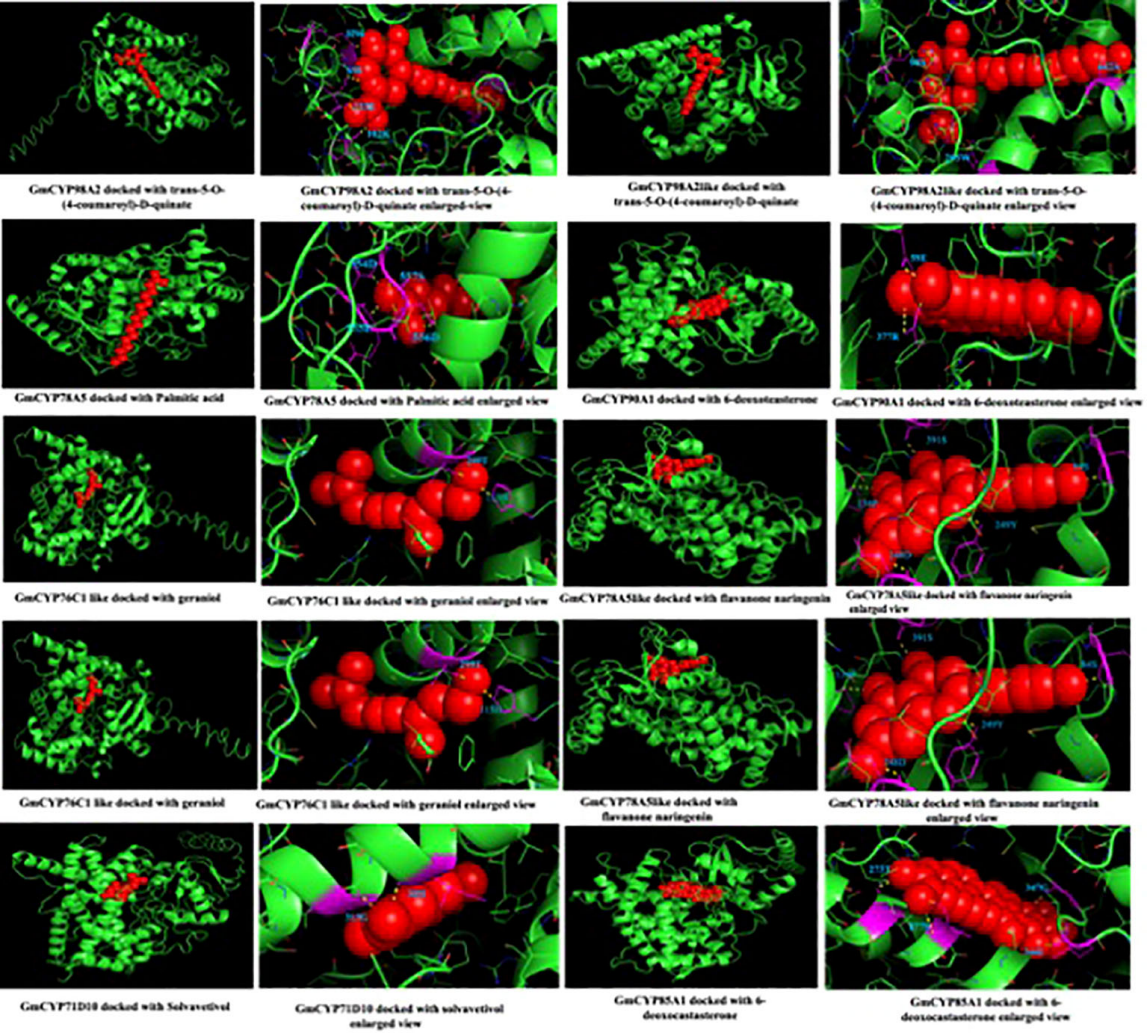
Interaction of *GmCYPs* with their substrate using molecular docking. The figure shows the modeled tertiary structures of *GmCYPs* (green colored) and their interaction with substrates (red colored). The enlarged view showing docked substrate with *GmCYPs*, amino acid residues forming polar bonds were highlighted (magenta color) and labelled.

**Figure 9:**
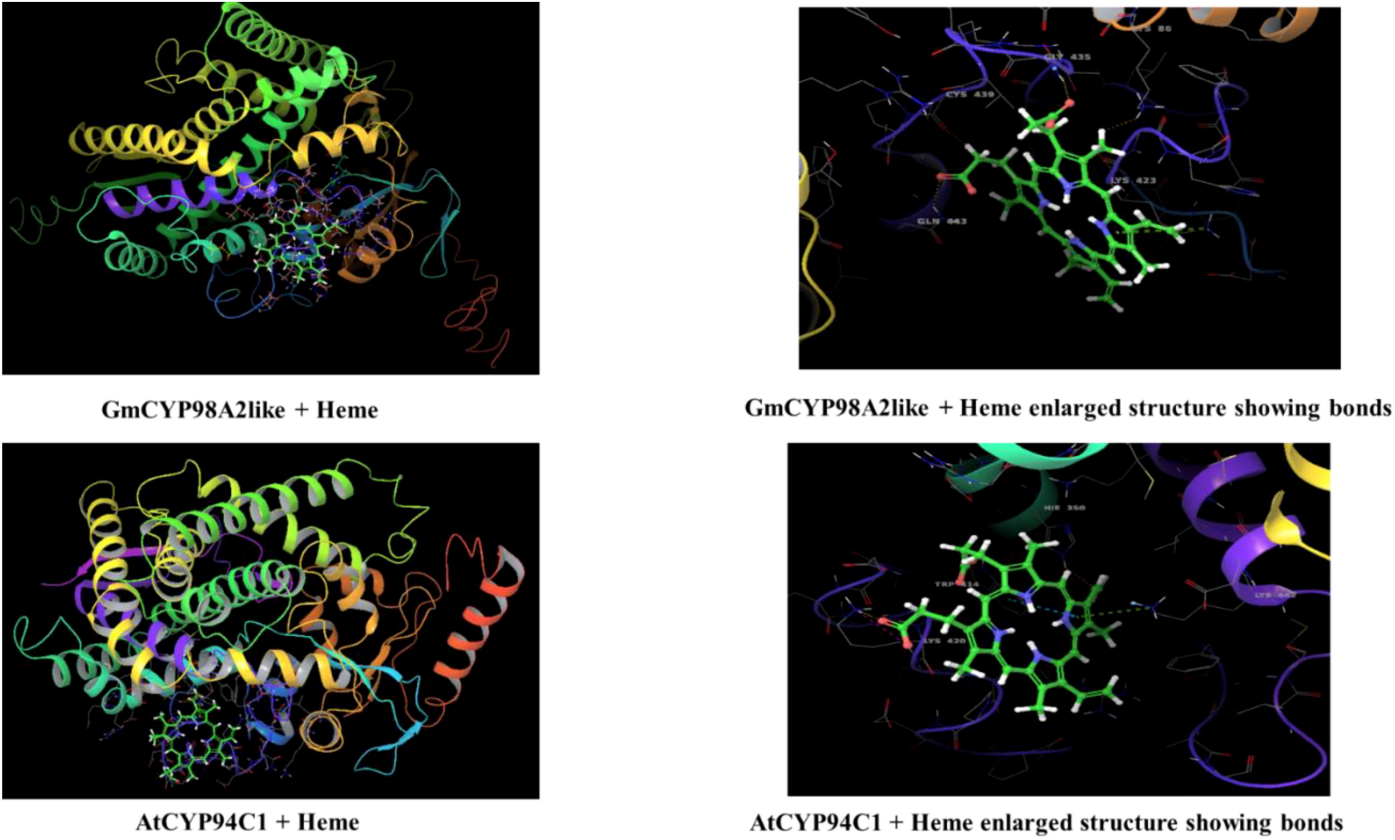
Interaction of GmCYP98A2like with heme using molecular docking. The figure shows the modeled tertiary structures of GmCYP and its interaction with the heme prosthetic group. The enlarged view showing amino acid residues (labelled and highlighted) of protein forming bonds with heme group. interaction of AtCYP94C1 with heme was also shown for the reference gene.

To study the enzymatic mechanism of *GmCYPs*, substrates of *GmCYPs* were retrieved from the literature survey and KEGG database. The interaction of 10 GmCYP with their specific substrate was examined through enzyme-substrate docking. The amino acid residues and polar bonds formed by substrate with *GmCYPs* catalyst were highlighted with magenta and yellow colors, respectively (**Figure 8**). The global energy score of *GmCYPs* and substrate interaction vary from -20 to -50, indicating their strong interaction (**Supplementary Table 5**). Earlier studies have reported that heme binding with CYP450s is essential for their enzymatic activities (Li et al., 2008). Therefore, molecular docking of a selected *GmCYPs* was performed with a heme ligand, and the interaction results were compared with AtCYP94C1 (**Figure 9**). The global energy score for GmCYP98A2 like protein and heme, was -3.392, while for AtCYP94C1 and heme was -2.341.

## 4 Discussion

Throughout their lifespan, plants confront a variety of stresses induced by both biotic and abiotic factors. To thwart themselves, plants have thrived in complex defence mechanisms that involve recognition of the particular stress and activating downstream signalling events. *CYPs* are versatile catalysts of a multi-gene superfamily, indulged in various growth, development-related processes and stress adaptation. CYPs reinforce the plant’s defence mechanism by regulating homeostasis, biosynthesis of secondary metabolites (defensive chemicals) and phytohormones and supplementing antioxidant compounds to scavenge reactive oxygen species (Glawisching, 2007; Li et al., 2013). CYPs are linked with abiotic stress and build up stress tolerance against biotic factors, viz. bacteria, viruses, fungi, insects and nematodes (Wang et al., 2001; Du Fall and Solomon, 2013; Irmisch et al., 2015). Plants are deprived of nutrients due to pest-infestation, which eventually results in a massive loss in crop yield. Although plants lack a fully developed immune system, they have developed sophisticated defence mechanisms to fend off intruders, constitutive defense and inducible defense mechanisms (Karban et al., 1997; Kessler and Baldwin, 2002). Constitutive defense involves physical barriers such as lignified cell walls, thick cuticles, wax formation and synthesis of defensive chemicals. In contrast, inducible defense leads to the biosynthesis of defensive chemicals, phytoalexins, volatile deterrents, cell wall strengthening and alternation in the expression pattern of defense-related genes and crosstalk network of phytohormones viz. jasmonic acid, ethylene, salicylic acid (Thomma et al., 1998; Walling, 2000; Rojo et al., 2003). CYP450s are indulged in aforesaid plant defense events during biotic stress. However, a few studies have been conducted on the role of CYPs against herbivory in different plant species, role of *GmCYPs* in plant defense against *S. litura-* herbivory remain elusive.

Therefore, to designate the function of CYPs in soybean and to rule out their involvement in defense against *S. litura*, a comprehensive analysis of *S. litura*- inducible 16 *GmCYPs* (reported in transcriptome analysis) was executed. Comprehending the evolutionary trend is crucial for understanding the diverse functions of a gene/gene family. Phylogenetic analysis accomplished using maximum-likelihood method suggests that CYPs from *G. max* and *M. truncatula* are closely related, indicating that they are orthologous species, which might have undergone changes during the course of evolution. The high bootstrap values further aid the accuracy and reliability of tree construction. The phylogenetic tree besides assessing level of evolution through instigating soybean genome may aid in comprehending the putative functions of soybean CYPs, drawing insight from functionally characterized CYPs in *Medicago* (Ma et al., 2014; Yang et al., 2022). In addition, Gene duplication analysis identified more orthologous pairs of *GmCYPs* with *MtrCYPs* than with *AtCYPs.* These findings indicate that the majority of *GmCYPs* are likely to be originated from gene duplication events. However, in all homologous gene pairs, Ka/Ks ratio was observed to be less than 1, indicating duplicated genes underwent purifying selection throughout their evolutionary history (Kondrashov et al., 2002; Chitkara et al., 2022).

The examination of *GmCYPs* physiological properties unveiled their hydrophilic, basic and stable nature. The subcellular localization of *GmCYPs* was primarily predicted to be localized in the endoplasmic reticulum, with minimal expression in other organelles, coinciding with earlier studies that also predicted their presence in the endoplasmic reticulum (Zhang and Li, 2017; Singh et al., 2021a). Examining exon-intron structure divulged that the structure of 16 *GmCYPs* is diverse with variable numbers of exons and introns, indicating pivotal evolutionary changes within the *G. max* genome. The gene *GmCYP89A2* lacks introns, while nine other *GmCYP*s contain a single intron, which speculated that these genes facilitate rapid transcriptional activity in response to adverse environmental conditions (Keshan et al., 2021; Wang et al., 2022). However, presence of fewer introns in *GmCYPs* indicates a lower level of evolutionary conservation (Paquette et al., 2000; Gorlova et al., 2014). The chromosomal mapping of *GmCYP*s showed that they were unevenly distributed across 13 soybean chromosomes, maximum on end positions. Genes with distal end position have been potential drivers of functional diversification while genes on proximal ends didn’t undergo recombination (Bulger and Groudine, 2011). The knowledge of the gene structure and chromosomal location can be used to design more efficient breeding strategies for developing soybean cultivars with desired traits. The conserved motifs and domains are indicative of protein potential roles. Identification of the P450 domain (conserved cytochrome family) and four conserved motifs (cysteine heme-iron ligand signature motif, PXRX motif, I-helix oxygen binding domain and K-helix region), confirming their membership in the cytochrome P450 superfamily and indicative of their potential functional roles and evolutionary conservation (Nelson et al., 1996; Li et al., 2007). The promoter region of a gene contains *cis*-elements that control the gene expression, which can vary during growth, development, and in response to environmental factors, in a plant organ-specific manner (Lata et al., 2014). *Cis*-acting elements in prediction analysis revealed the appearance of many light-responsive elements indicating their role in light signalling and photosynthesis. The presence of phytohormone-responsive cis-elements, including methyl-jasmonate, salicylic acid, gibberellin, and abscisic acid in the promoter region of *GmCYP*s reflecting induction of these genes under plant hormone pathway and innate immunity. In addition, the presence of low-temperature-responsive elements, drought-responsive elements, anaerobic condition-responsive elements, flavonoid biosynthesis regulating elements, defense-responsive elements, which implies that these genes could respond to and adapt to stress (Keshan et al., 2021). Earlier reports support the cis-acting element based regulation of the *GmCYP*s under stress, which can act as potential targets for crop improvement (Wang et al., 2022; Yang et al., 2022). Furthermore, various types of TFBSs were identified in promoters of *GmCYPs*. These TFs play crucial roles in the transcriptional regulation of diverse abiotic and biotic stress responses along with developmental processes (Nakano et al., 2006; Lijavetzk,y et al., 2003). The previous reports endorsed the fact that TFs have importance against environmental stress (Belamkar et al., 2014; Cui et al., 2019). However, prediction of 94 putative potential miRNA target sites in 16 *GmCYPs* reveals implications of miRNA-mediated stress regulation in soybean through post-transcriptional mechanism (Bhatia et al., 2019).

The study of mRNA abundance measurements for a family of genes in an organ-specific manner will enable the identification of the genes engaged in regulatory or development processes specific to a given plant-organ type. Overall, the RNA-seq expression data analysis provided valuable insights into the pattern of expression specific to certain plant-organs of the listed *GmCYPs*, which could aid in understanding their functional roles in soybean growth and development (Ma et al., 2014; Saxena et al., 2023). The *GmCYPs* gene expression in different organs showed their functional divergence (Lopes-Caitar et al., 2013). Nevertheless, a significant number of *GmCYPs* showed minimal or no expression in various plant-organs, suggesting their involvement in alternative process.

Protein-protein interaction analysis (PPI) was executed with the STRING database to infer the intricate interplay of *GmCYPs* with other soybean proteins, enrichment analysis and the network. The result deduced that interactions were diverse, and 8 out of 16 *GmCYPs* proteins interact with other proteins involved in the phenylpropanoid pathway, secondary metabolite synthesis, pathogen responsive pathway and flavonoid biosynthesis, reported to defend the plant from biotic stress ranges from physical to chemical barrier, including signalling pathways (Dixon et al., 2002; Shah and Smith, 2020). They are also inferred to interact with proteins responsive to heavy metal tolerance, brassinolide biosynthesis and oxidative stress tolerance; predict their role in stress tolerance. However, the results of PPI are based on prediction and should be interpreted with caution. It would be necessary to conduct additional empirical investigations and experiments to determine whether these connections are physiologically significant.

Gene expression analysis during *S.litura*-infestation and wounding has been done in various plants like *Arabidopsis*, in chickpea, and soybean etc. to concede intricate changes in phytohormone and signalling pathway, attributing their role in plant defense against pest invasion. Earlier researches uphold the fact that, CYPs are involved in resistance against pest invasion. Resistance to *Myzus persicae* (green peach aphid) was regulated by camalexin synthesis by CYP family gene (Prince et al., 2014). Suppression of CYP hydroxylase in *N. tabaccum* enhanced the resistance to *Myzus nicotianae* and *CYP79D* was also reported to enhance aldomixes in *Populus* sp. (Wang et al., 2001; Irmisch et al., 2015). However, gene expression changes during mechanical wounding were different from herbivory. These findings revealed that herbivory and wounding had distinct transcript patterns since along with cell wall disruption, herbivory also involves the ingestion of insect-derived elicitors. Previous reports have also shown a difference between the expression patterns during insect attacks and mechanical wounding (Reymond et al., 2000).

A complex interplay of phytohormones, such as jasmonate, ethylene and salicylic acid, serves as a crucial plant signal, evoked after insect pest or pathogen attack and activates defence genes. Plant pests mostly activate jasmonate/ethylene signalling pathway and also regulate salicylic acid pathway (Reymond et al., 2004). However, salicylic and jasmonate hormones induce the expression of different genes and act antagonistically. In order to acknowledge the role of these phytohormones, gene expression analysis of *GmCYP*s was examined upon their foliar treatment on plant. Alternation in *GmCYPs* expression level on treatment with these hormones demonstrate their role in regulation of *GmCYPs*. CYPs are known to regulate jasmonate signalling pathway as alternation in JA gene expression was observed upon herbivory, wounding and pathogen attack (Blée, 2002; Bari and Jones, 2009). *AtCYP82C2* modulates defense against *Botrytis cinerea*, and *CYP82D2* regulates defense gene expression against *V. dahlia* by regulating the expression of jasmonate-induced defense-related genes (Reviewed by Singh et al., 2008).

The tertiary structure of selected 16 *GmCYPs* was predicted to study the interaction between *GmCYPs*, heme and also their substrates, and this revealed a variation in the modelled structures of *GmCYPs*, primarily because of the variation in the number and sequences of the amino acids present. Their different structural configurations could enable them to serve a particular function. However, the characterization and localization of evolutionarily conserved motifs in 16 *GmCYPs* strongly uphold the identity of *GmCYPs* as a part of CYP450 superfamily. Besides, the interaction of the heme cofactor with GmCYP, which is essential for their catalytic activity, was examined by selecting heme-binding region residues for generating a receptor grid. The global energy score of GmCYP98A2-like, docked with heme, was compared to AtCYP94C1, an enzyme involved in the jasmonate biosynthesis pathway (Heitz et al., 2012). Protein-protein docking was performed to examine the interaction between *GmCYPs* enzymes and their substrates. Global energy score for interaction between substrate and GmCYP varied from -25 to -50 but was comparable to receptor COX-2 and chalcone (Shilpa and Varalakshmi Devi, 2017). Evaluation of enzyme-substrate interaction showed the presence of polar bonds at the active sites of enzyme and substrate, which are stronger bonds that lower the activation energy of the enzyme-substrate complex and enhance the feasibility of reaction and product formation.

*GmCYP78A5*, *GmCYP78A3*, *GmCYP78A5-like*, and *GmCYP76C1-like* have induced gene expression on *S. litura-*infestation and methyl-jasmonate treatment, while *GmCYP78A5-like* and *GmCYP76C1-like* are also induced upon mechanical wounding. GmCYP78A5-like protein interaction was predicted with Alpha-ketoglutarate-dependent dioxygenases involved in flavonoid and ethylene biosynthesis. This protein also showed the presence of methyl jasmonate-responsive cis-element in its promoter region. *GmCYP78A5-like* is Flavonoid 3’-monooxygenase that catalyse formation of flavanone naringenin that showed detrimental effects on insect pests (Goławska et al., 2014). *GmCYP76C1-like* have methyl-jasmonate responsive *cis*-element, interacts with non-specific serine/threonine protein kinase; CYP b5-like steroid binding domain-containing protein. *GmCYP76C1-like*, known as geraniol 8-hydroxylase produces geraniol, which has repellent properties; availing as a natural pest control chemical with low toxicity (Chen and Viljoen, 2010). *GmCYP78A5* xenobiotic monooxygenase synthesizes palmitic acid (PA), which metabolises xenobiotics and reduces the incidence of soil-borne diseases (Carta et al., 2017). *GmCYP78A5* has been predicted to interact with Delta(7)-sterol-C5(6)-desaturase involved in the biosynthesis of sterols and methyl jasmonate responsive *cis*-element (Feng et al., 2017; Saini and Kumar, 2019). Further, functional characterization of these genes in soybean will confirm their roles in insect resistance and will be used in molecular breeding and genetic engineering.

## 5 Conclusion

CYPs are multifunctional catalysts involved in various physiological processes of plants and contribute significantly to plant defense through the biosynthesis of hormones, secondary metabolites, fatty acids and cell wall components. Recent characterization of various CYPs has revealed their potential to exploit in agricultural improvement to create stress-tolerant plants. Even though the entire soybean genome sequence has been accessible since 2010 and the significance of CYP450s in plant metabolic pathways have long been understood, only a few P450s in soybean have been evaluated to characterize their role in herbivory. In our study, an extensive analysis of biotic stress-responsive *GmCYPs* was performed to deduce their role in soybean defense against *S. litura* herbivory. The phylogenetic study and motif analysis revealed their relatedness to other *GmCYPs* from model plants. Identification of biotic stress-responsive cis-element in the promoter region of *GmCYPs*, PPI analysis and their differential expression upon *S. litura* infestation, and treatment of signalling compounds proposed their significant function in plant defense. Further, structural characterization by interaction study between *GmCYPs* and heme cofactor and enzyme-substrate interactions confirmed their identity as heme-binding proteins, and allowed us to infer thermodynamic feasibility of reactions. *GmCYP78A5like*, *GmCYP76C1* are concluded as potential candidates involved in soybean-*S. litura* interaction. The findings of this study can be expanded to confirm the underlying mechanism of *GmCYPs* in plants during *S. litura* -infestation. Further *in vivo* functional and structural characterization of these genes employing techniques such as genome editing (overexpression-RNAi) and traditional genetic breeding to confirm their function in plant defense is highly recommended.

## Data availability statement

The original contributions presented in the study are included in the article/**Supplementary Material**. Further inquiries can be directed to the corresponding authors.

## Author contributions

AS and IS conceptualized and supervised the study. MY and RP performed the experiments, collected the data and analyzed the results. MY, RP and AnR wrote the manuscript. AS, IS, AC and AmR critically reviewed the manuscript. AS, IKS, AC and AmR contributed to editing and visualization. AS, IS and AmR contributed to the formal analysis. AS, IS and AmR contributed to funding acquisition. IS and AS contributed to resources. All authors contributed to the article and approved the submitted version.
