# Supplementary material for: Comprehensive and evolutionary analysis of Spodoptera litura-inducible Cytochrome P450 monooxygenase gene family in Glycine max elucidate their role in defense
Source: Front Plant Sci. 2023 Nov 2;14:1221526. doi: 10.3389/fpls.2023.1221526 (PMC10654349; doi:10.3389/fpls.2023.1221526)
Supplement: Supplementary file 1 [file DataSheet_1.docx]

**Comprehensive and evolutionary analysis of *Spodoptera litura*-inducible Cytochrome P450 monooxygenase gene family in *Glycine max* elucidate their role in defense**

Manisha Yadav^1,2^, Ruby Panwar^1,3^, Anjana Rustagi^3^, Amrita Chakraborty^4^, Amit Roy^5^*, Indrakant K Singh^6^* and Archana Singh^1,2,7^*

^1^Department of Botany, Hansraj College, University of Delhi, Delhi, 110007, India

^2^J C Bose Center for Plant Genomics, Hansraj College, University of Delhi, Delhi, India. ^3^Department of Botany, Gargi College, University of Delhi, Delhi, 110007, India

^4^ EVA 4.0 Unit, Faculty of Forestry and Wood Sciences, Czech University of Life Sciences Prague, Kamýcká 129, 165 21 Praha 6 – Suchdol.

^5^Forest Molecular Entomology Lab, EXTEMIT-K, EVA 4.0, Faculty of Forestry and Wood Sciences, Czech University of Life Sciences Prague, Kamýcká 129, 165 21 Praha 6 – Suchdol.

^6^Molecular Biology Research Lab, Department of Zoology, Deshbandhu College, University of Delhi, Kalkaji, New Delhi, 110019, India;

^7^Delhi School of Climate Change and Sustainability, Institution of Eminence, Maharishi Karnad Bhawan, University of Delhi, Delhi, India

**Supplementary figures**

**Supplementary Figure: 1** Heat map showing predicted subcellular location of 16 *GmCYPs* using Deep-Loc.

**Supplementary Figure: 2** Gene structures (exon/intron) of 16 *GmCYPs*. The gene structure analysis performed using Gene Structure Display server GSDS2.0 showing the distribution of exon-intron in *GmCYPs*. The exons are represented by brown, upstream/downstream are presented by red, and introns are represented by solid black line. The size and relative position of exons can be approximated based on the scale at the bottom.

**Supplementary Figure: 3** Chromosomal distributions of 16 *GmCYPs.* 16 *GmCYPs* were mapped onto *Glycine max* chromosomes based on their physical location and chromosomes are represented in different colours.

**Supplementary Figure: 4** Protein domain and motifs of selected 16 GmCYPs. (A) Functional protein domain analysis of GmCYPs showing p450 domain in each CYP analysed using Pfam and NCBI conserved domain search tool. (B) Different putative conserved motifs and related positions in each GmCYP are represented by colored boxes. (C) Logo plots of different motifs was analysed by MEME suite.

**Supplementary Figure: 5** Prediction of conserved motifs in 16 GmCYPs by Multiple sequence alignment. CYPs proteins have four highly conserved motifs shown in (A) the heme-binding region (FXXGXRXCXG) in C-terminus, (B) PXRX motif, (C) K-helix region (EXXR) and (D) I-helix oxygen-binding domain. Heme binding region is for heme binding and (EXXR and PXRX) is crucial for locking the heme pocket into position, thus ensuring the stability of the core structure.

**Supplementary Figure: 6** Bar graph showing TF binding sites**-**Transcription families found in promoter region of *GmCYPs*

**Supplementary Figure: 7** Protein-protein interactions (PPI) network of GmCYPs (which showed interaction with other stress- responsive proteins). Protein interaction of different GmCYPs (represented in red) with other *Glycine max* proteins was analyzed using string database.

**Supplementary Figure: 8** Pictures showing Ramachandran plot analysis of 3-D predicted structures of GmCYPs using i-tasser.

**
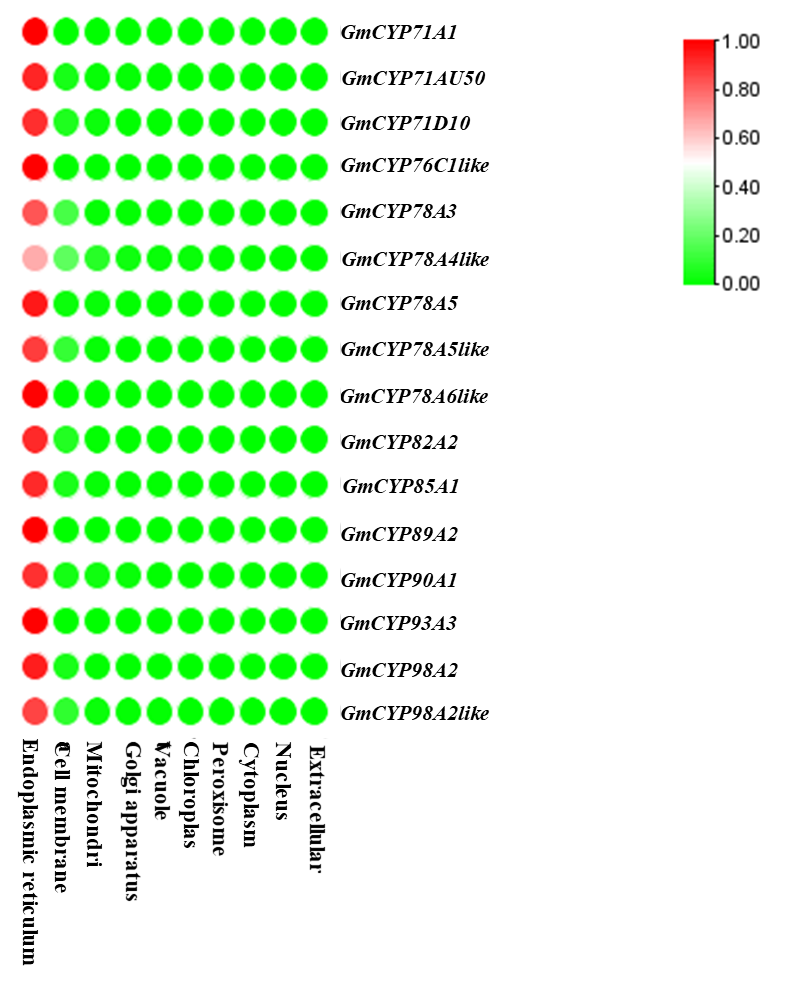
**

Supplementary Figure 1

Supplementary Figure 2

**
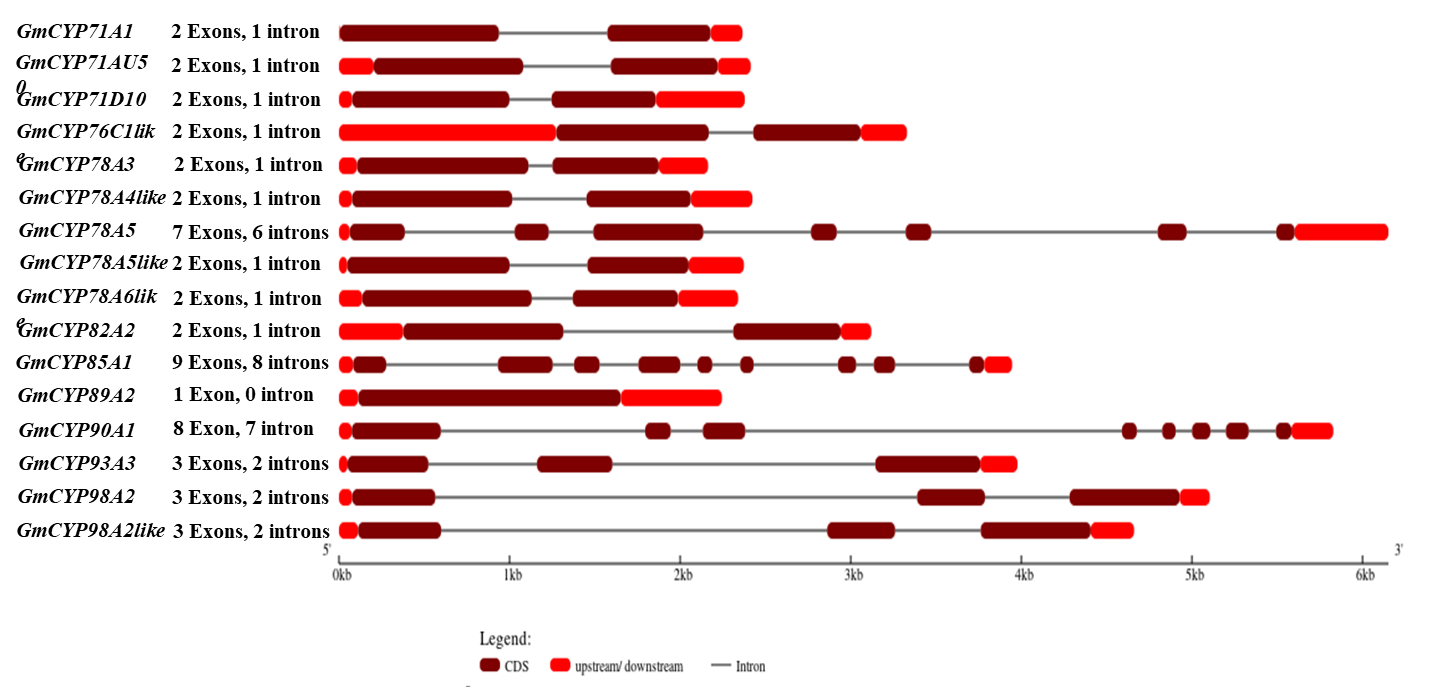
**

Supplementary Figure 3

**
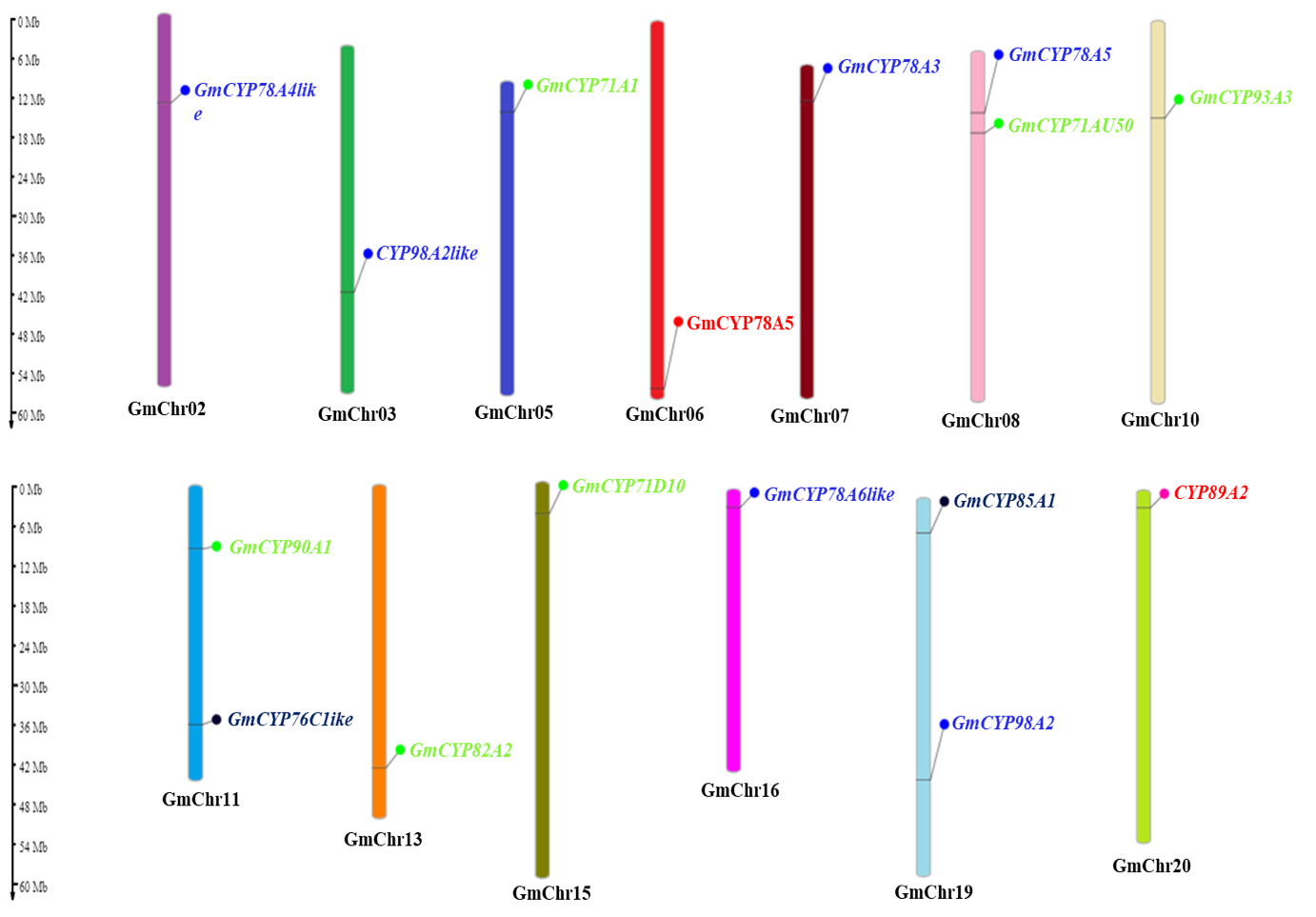
**

Supplementary Figure 4

**
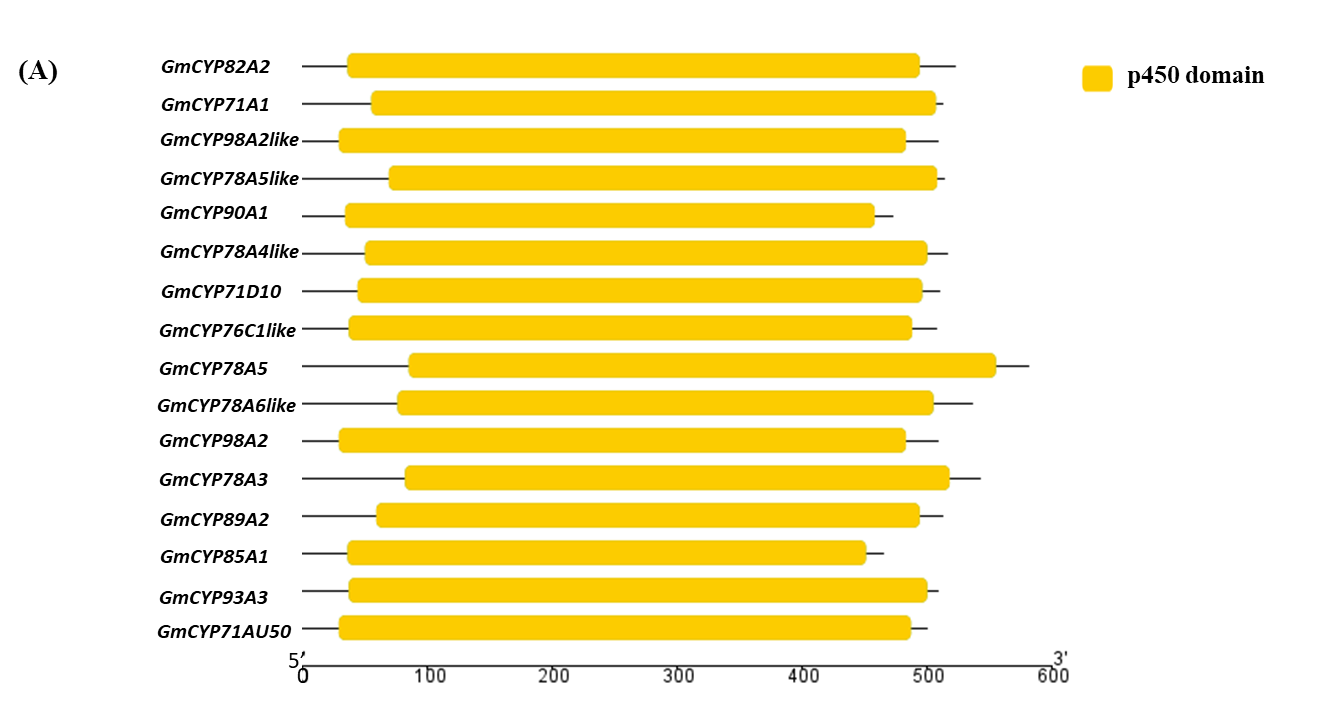
**

**
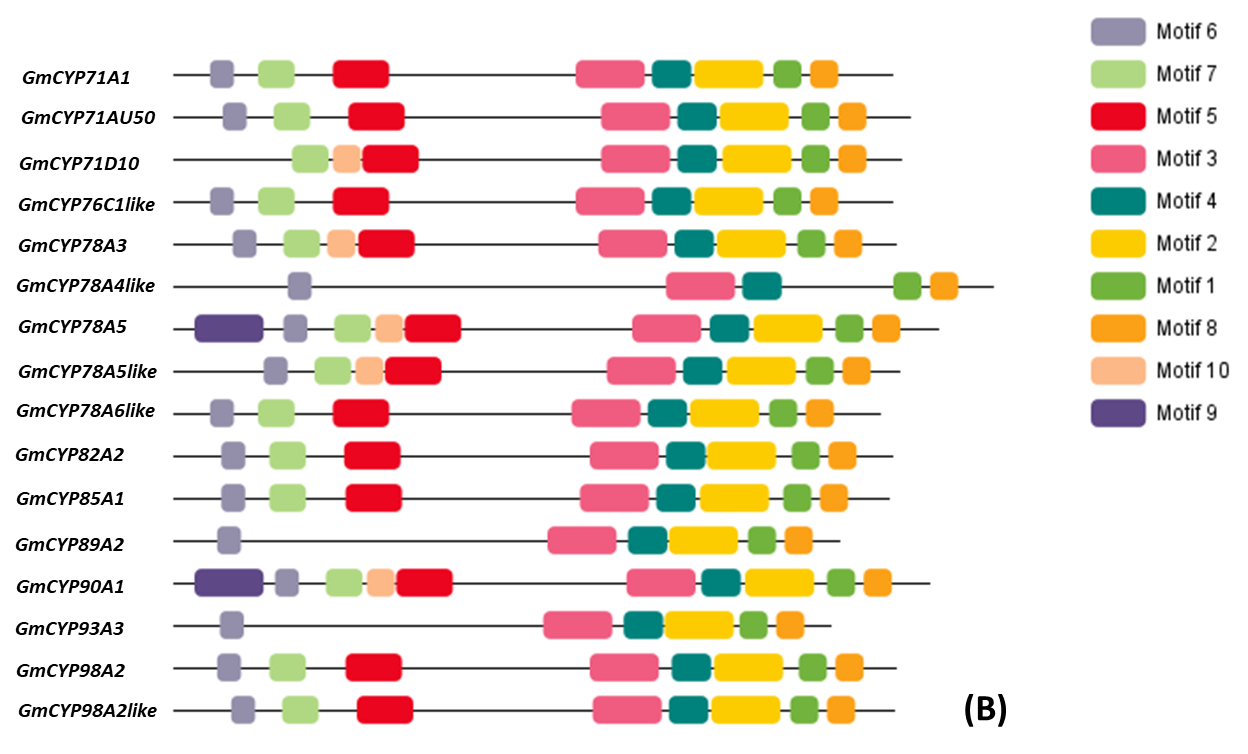
**

**
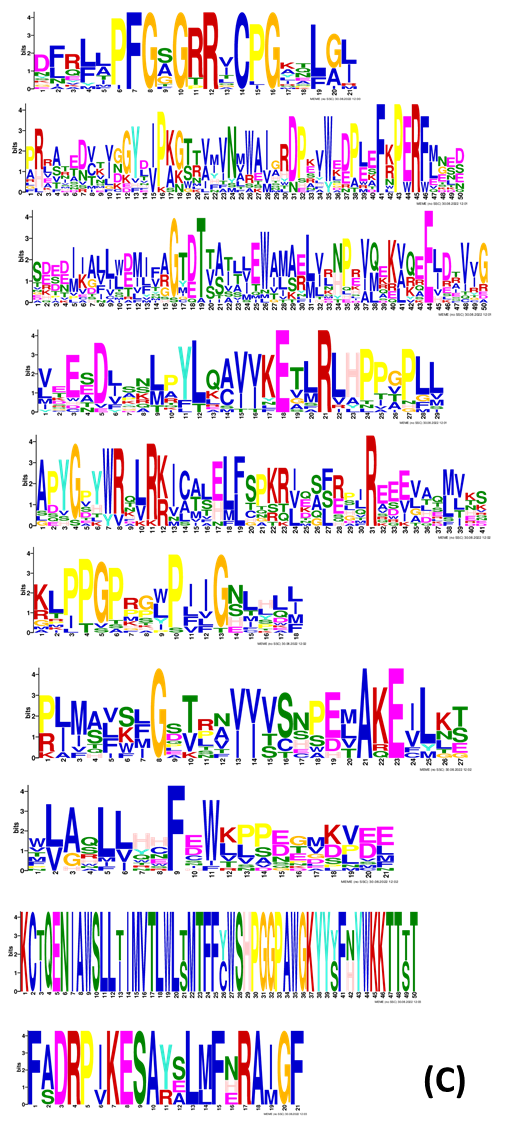
**

Supplementary Figure 5

Supplementary Figure 6

**
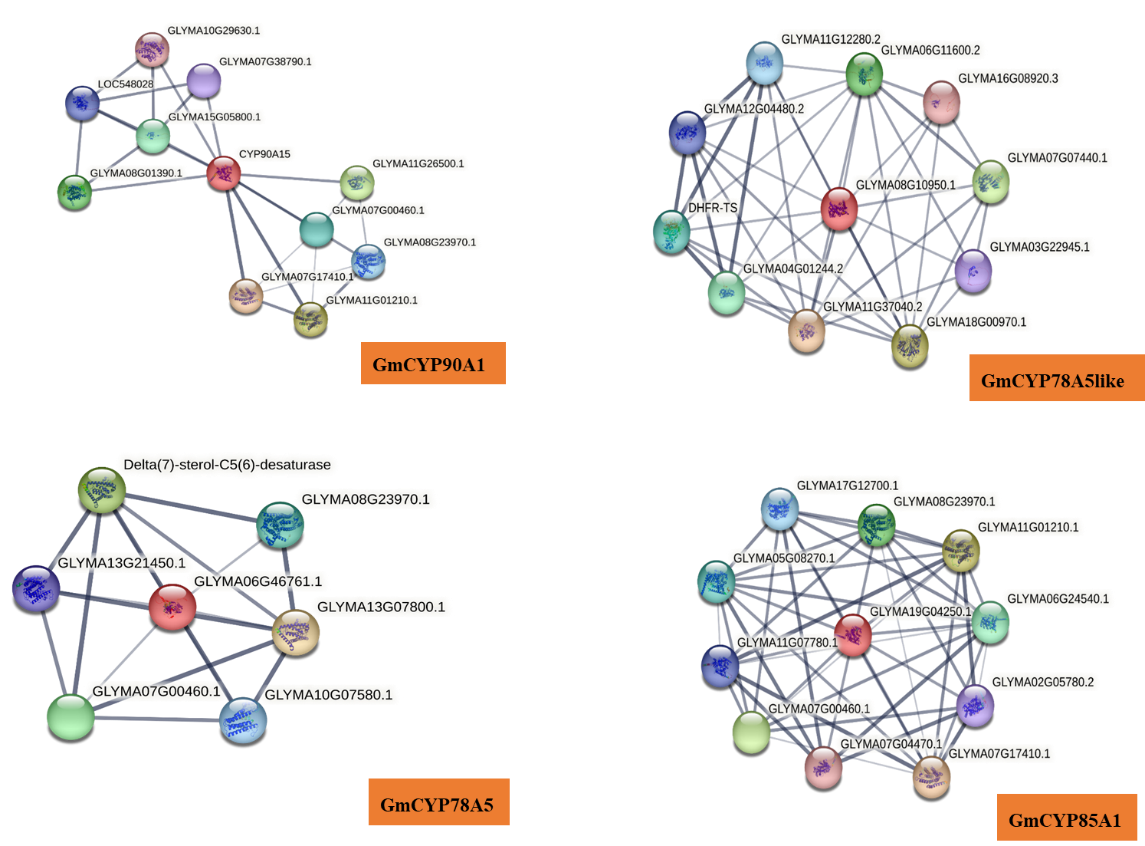

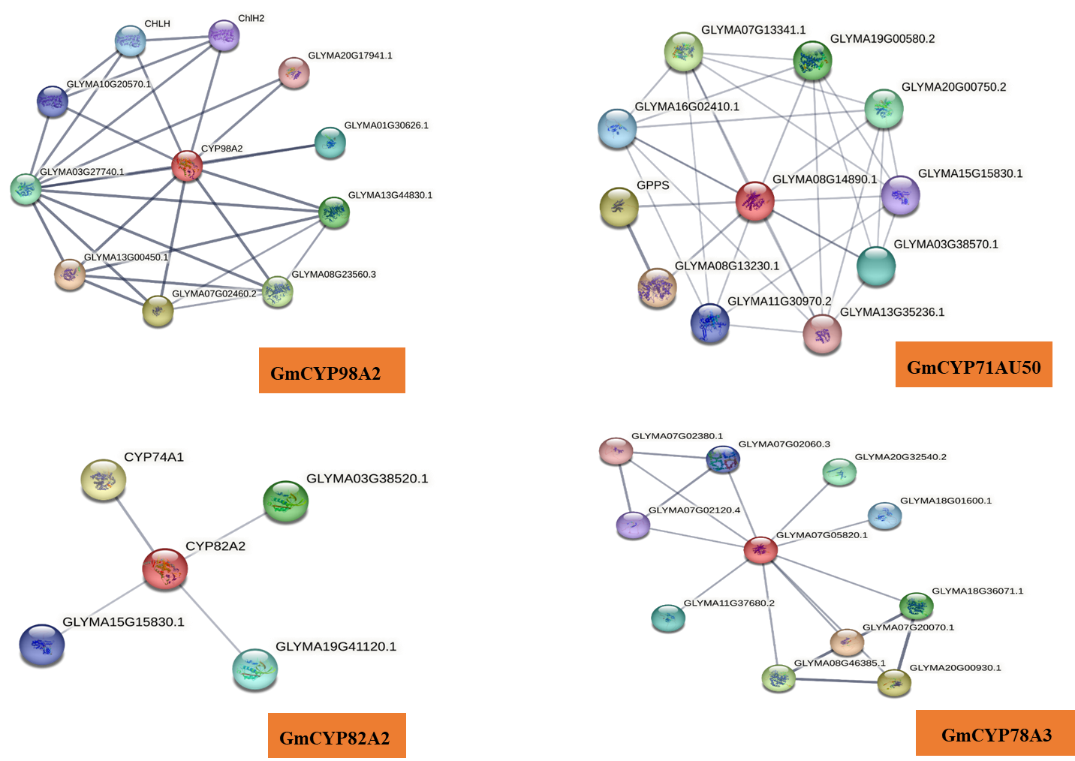
**

Supplementary Figure 7

Supplementary Figure 8

Supplementary Tables

| **Gene Name** | **Phytozome ID** | **Uniprot ID** | **Peptide sequences** | **Molecular weight (kDa)** | **pI** | **Instability index** | **Aliphatic Index** | **GRAVY** |
| --- | --- | --- | --- | --- | --- | --- | --- | --- |
| GmCYP98A2 | Glyma.19G126000.1 | [O48922](https://www.uniprot.org/uniprotkb/O48922/entry) | 510 | 57.674 | 8.58 | 36.45, Stable | 93.28 | -0.19 |
| GmCYP82A2 | Glyma.13G285300.1 | [O81972](https://www.uniprot.org/uniprotkb/O81972/entry) | 523 | 58.715 | 6.54 | 36.18, Stable | 100.06 | -0.059 |
| GmCYP78A4like | Glyma.02G119600.1 | [I1JEG7](https://www.uniprot.org/uniprotkb/I1JEG7/entry) | 517 | 58.316 | 6.96 | 35.84, Stable | 94.4 | -0.002 |
| GmCYP98A2like | Glyma.03G122000.1 | [I1JN04](https://www.uniprot.org/uniprotkb/I1JN04/entry) | 510 | 57.706 | 8.72 | 36.28, Stable | 92.32 | -0.198 |
| GmCYP71A1 | Glyma.05G042600.1 | [I1K057](https://www.uniprot.org/uniprotkb/I1K057/entry) | 513 | 58.483 | 9.01 | 36.11, Stable | 91.58 | -0.108 |
| GmCYP78A5 | Glyma.06G310800.1 | [K7KYE8](https://www.uniprot.org/uniprotkb/K7KYE8/entry) | 582 | 65.343 | 8.06 | 38.15, Stable | 86.95 | -0.129 |
| GmCYP78A3 | Glyma.07G052300.1 | [I1KHS1](https://www.uniprot.org/uniprotkb/I1KHS1/entry) | 543 | 61.033 | 8.9 | 33.48, Stable | 89.24 | -0.06 |
| GmCYP78A5like | Glyma.08G104100.1 | [I1KS17](https://www.uniprot.org/uniprotkb/I1KS17/entry) | 515 | 57.914 | 9.01 | 39.69, Stable | 96.75 | 0.021 |
| GmCYP71AU50 | Glyma.08G140500.1 | [I1KT85](https://www.uniprot.org/uniprotkb/I1KT85/entry) | 501 | 57.199 | 8.69 | 42.93, Unstable | 95.92 | -0.204 |
| GmCYP93A3 | Glyma.10G092500.1 | [A0A0R0HYP7](https://www.uniprot.org/uniprotkb/A0A0R0HYP7/entry) | 510 | 57.967 | 6.67 | 42.3, Unstable | 94.62 | -0.187 |
| GmCYP76C1like | Glyma.11G108300.1 | [I1LJ26](https://www.uniprot.org/uniprotkb/I1LJ26/entry) | 508 | 54.135 | 7.14 | 32.24, Stable | 102.11 | 0.030 |
| GmCYP90A1 | Glyma.11G228900.1 | [K7LRQ4](https://www.uniprot.org/uniprotkb/K7LRQ4/entry) | 473 | 53.942 | 9.27 | 39.8, Stable | 93.18 | -0.137 |
| GmCYP78A6like | Glyma.16G021200.1 | [I1MKF4](https://www.uniprot.org/uniprotkb/I1MKF4/entry) | 537 | 60.415 | 7.66 | 32.05, Stable | 93 | 0.038 |
| GmCYP85A1 | Glyma.19G033900.1 | [I1N6E7](https://www.uniprot.org/uniprotkb/I1N6E7/entry) | 466 | 53.375 | 9 | 34.85, Stable | 87.83 | -0.185 |
| GmCYP89A2 | Glyma.20G018800.1 | [A0A0R4J634](https://www.uniprot.org/uniprotkb/A0A0R4J634/entry) | 513 | 59.257 | 8.59 | 45.82, Unstable | 97.79 | -0.13 |
| GmCYP71D10 | Glyma.15G050300.1 | [O48923](https://www.uniprot.org/uniprotkb/O48923/entry) | 511 | 58.462 | 8.59 | 45.98, Unstable | 92.67 | -0.222 |

Supplementary Table 1A: List of 16 *GmCYPs* and their physiochemical properties.

| Gene duplication | pairs |  |  |  |
| --- | --- | --- | --- | --- |
| **Gene 1** | **Gene 2** | **Ka** | **Ks** | **Ka/Ks** |
| NP_850337.1 | XP_003521103.1 | 2.3615 | 0.1167 | 0.049418 |
| NP_182189.1 | XP_003528776.1 | 2.1612 | 0.258 | 0.119378 |
| NP_001190156.1 | XP_003528776.1 | 2.7056 | 0.2473 | 0.091403 |
| NP_182189.1 | XP_003548215.1 | 2.1431 | 0.2702 | 0.126079 |
| NP_001190156.1 | XP_003548215.1 | 2.4934 | 0.2509 | 0.100626 |
| XP_006582383.1 | XP_039688169.1 | 0.441558 | 0.14702 | 0.332959 |
| XP_003528776.1 | XP_003627517.1 | 1.3151 | 0.1552 | 0.118 |
| XP_003531193.1 | XP_013446567.1 | 0.9283 | 0.1396 | 0.1504 |
| XP_006588911.1 | XP_013442456.1 | 1.6076 | 0.2008 | 0.1249 |
| XP_003538460.1 | XP_003600878.1 | 0.7166 | 0.1094 | 0.1527 |
| XP_003548215.1 | XP_003627517.1 | 1.15 | 0.1488 | 0.1294 |
| XP_003554965.1 | XP_013451102.1 | 0.5204 | 0.0617 | 0.1185 |
| XP_003554965.1 | XP_013447676.1 | 0.7869 | 0.0871 | 0.1107 |
| NP_001235563.1 | XP_003521103.1 | 0.1406 | 0.018 | 0.1279 |
| NP_001304524.1 | XP_003528776.1 | 0.9706 | 0.1598 | 0.1646 |
| XP_003548215.1 | XP_003528776.1 | 0.3131 | 0.0489 | 0.156 |
| XP_003538551.1 | XP_003531193.1 | 0.9682 | 0.1633 | 0.1687 |
| XP_003524168.1 | XP_003531193.1 | 0.2291 | 0.036 | 0.1572 |
| XP_003538460.1 | XP_014625807.1 | 0.1264 | 0.0348 | 0.2757 |
| XP_003538460.1 | XP_003519393.1 | 0.6447 | 0.0911 | 0.1413 |
| XP_003538460.1 | XP_003545232.1 | 0.6598 | 0.0897 | 0.1359 |
| XP_003548215.1 | NP_001304524.1 | 1.0612 | 0.172 | 0.1621 |
| XP_003548215.1 | XP_003528776.1 | 0.3131 | 0.0489 | 0.156 |
| XP_003544008.3 | XP_003554965.1 | 0.1348 | 0.0245 | 0.1818 |
| NP_001241912.2 | XP_003554965.1 | 0.4847 | 0.0727 | 0.15 |

Supplementary Table 1 B. Gene duplication rates for GmCYPs genes showing non-synonymous (Ka) and synonymous (Ks) substitution rates and their ration (Ka/Ks)

| **Gene Name** | **Gene Id** | **Gene length** | **CDS length** | **Chromosome localisation** | **Strand** | **Start point** | **End point** |
| --- | --- | --- | --- | --- | --- | --- | --- |
| GmCYP98A2 | 606506 | 5105 | 1530 | 19 | Reverse | 38430096 | 38435201 |
| GmCYP82A2 | 100798546 | 3121 | 1569 | 13 | Forward | 38620639 | 38623760 |
| GmCYP78A4like | 100804462 | 2424 | 1551 | 2 | Forward | 11775035 | 11777459 |
| GmCYP98A2like | 100811080 | 4661 | 1530 | 3 | Reverse | 33466243 | 33470904 |
| GmCYP71A1 | 100781909 | 2366 | 1539 | 5 | Reverse | 3813745 | 3816111 |
| GmCYP78A5 | 100809563 | 6152 | 1746 | 6 | Forward | 49935879 | 49942031 |
| GmCYP78A3 | 100798388 | 2164 | 1629 | 7 | Forward | 4557778 | 4559942 |
| GmCYP78A5like | 100817944 | 2374 | 1545 | 8 | Forward | 7993316 | 7995690 |
| GmCYP71AU50 | 100796116 | 2414 | 1503 | 8 | Forward | 10748125 | 10750539 |
| GmCYP93A3 | 100805931 | 3978 | 1530 | 10 | Reverse | 12875705 | 12879683 |
| GmCYP76C1like | 100807166 | 3330 | 1524 | 11 | Reverse | 8261600 | 8264930 |
| GmCYP90A1 | 100795125 | 5829 | 1419 | 11 | Forward | 32424577 | 32430406 |
| GmCYP78A6like | 100815292 | 2340 | 1611 | 16 | Forward | 1996083 | 1998423 |
| GmCYP85A1 | 100805958 | 3946 | 1398 | 19 | Reverse | 4363980 | 4367926 |
| GmCYP89A2 | 100795683 | 2245 | 1539 | 20 | Forward | 1944212 | 1946457 |
| GmCYP71D10 | 606548 | 2379 | 1533 | 15 | Forward | 3968864 | 3971243 |

Supplementary Table 2: showing information about chromosomal location of *GmCYPs* genes.

| **Protein** | **C-score** | **Estimated TM** | **Estimated Z score** | **Coverage** | **Template** |
| --- | --- | --- | --- | --- | --- |
| GmCYP82A2 | -0.71 | 0.62±0.14 | 9.0±4.6Å | 0.89 | Cinnamate 4-hydroxylase (C4H1) from *Sorghum bicolor*, **6vbyA** |
| Gm78A4 like | -1.41 | 0.54±0.15 | 10.7±4.6Å | 0.88 | CYP76AH1 from *Salvia miltiorrhiza,* 5ylwA |
| GmCYP71D10 | -0.99 | 0.59±0.14 | 9.6±4.6Å | 0.90 | CYP76AH1 from *Salvia miltiorrhiza,* 5ylwA |
| GmCYP85A1 | 0.22 | 0.74±0.11 | 6.7±4.0Å | 0.92 | Crystal structure of CYP90B1 in complex with brassinazole at, 6a17A |
| GmCYP90A1 | 0.15 | 0.73±0.11 | 6.8±4.1Å | 0.93 | Crystal structure of CYP90B1 in complex with brassinazole at, 6a17A |
| 93A3 | -0.59 | 0.64±0.13 | 8.7±4.5Å | 0.90 | CYP76AH1 from *Salvia miltiorrhiza,* 5ylwA |
| 98A2 | -0.27 | 0.68±0.12 | 8.0±4.4Å | 0.90 | CYP76AH1 from *Salvia miltiorrhiza,* 5ylwA |
| 98A2like | -0.46 | 0.65±0.13 | 8.4±4.5Å | 0.90 | CYP76AH1 from *Salvia miltiorrhiza,* 5ylwA |
| 89A2 | -0.50 | 0.65±0.13 | 8.5±4.5Å | \| 0.90 \|  \| \| --- \| --- \| | CYP76AH1 from *Salvia miltiorrhiza,* 5ylwA |
| 78A6 like | -0.81 | 0.61±0.14 | 9.3±4.6Å | 0.93 | *S. cerevisiae* CYP51 COMPLEXED WITH VT-1129, 7ryxA |
| 76C1like | -0.73 | 0.62±0.14 | 9.0±4.6Å | 0.90 | CYP76AH1 from *Salvia miltiorrhiza,* **5ylwA** |
| 71AU50 | -0.10 | 0.70±0.12 | 7.5±4.3Å | 0.92 | CYP76AH1 from *Salvia miltiorrhiza,* **5ylwA** |
| 78A5like | -1.57 | 0.52±0.15 | 11.1±4.6Å | 0.86 | CYP76AH1 from *Salvia miltiorrhiza,* **5ylwA** |
| 78A3 | -1.73 | 0.50±0.15 | 11.6±4.5Å | \| 0.92 \|  \| \| --- \| --- \| | *S. cerevisiae* CYP51 COMPLEXED WITH VT-1129, 7ryxA |
| 71A1 | -0.96 | 0.59±0.14 | 9.6±4.6 Å | 0.89 | CYP76AH1 from *Salvia miltiorrhiza*, 5ylwA |
| 78A5 | -1.35 | 0.55±0.15 | 10.8±4.6Å | 0.78 | CYP76AH1 from *Salvia miltiorrhiza* **5ylwA** |

Supplementary Table 3: The values of C score, estimated TM, Z score and template for three dimensional structure prediction of GmCYPs.

| **Name** | **Enzyme** | **Docking Score** | **Substrate** |  |
| --- | --- | --- | --- | --- |
| CYP 98A2 | [5-O-(4-coumaroyl)-D-quinate 3'-monooxygenase](https://www.genome.jp/entry/1.14.14.96) | **-40.47** | [trans-5-O-(4-coumaroyl)-D-quinate](https://www.brenda-enzymes.de/ligand.php?brenda_ligand_id=44894) |  |
| CYP 98A2 like | 5-O-(4-coumaroyl)-D-quinate 3'-monooxygenase | **-34.34** | [trans-5-O-(4-coumaroyl)-D-quinate](https://www.brenda-enzymes.de/ligand.php?brenda_ligand_id=44894) |  |
| CYP 78A5 | Xenobiotic monooxygenase | **-23.1** | Palmitic acid |  |
| CYP 78A5 like | Flavonoid 3'-monooxygenase | **-44.16** | flavanone naringenin |  |
| CYP 93A3 | 3,9-dihydroxypterocarpan 6A-monooxygenase | **-35.74** | [(6aR,11aR)-3,9-dihydroxypterocarpan](https://www.brenda-enzymes.org/ligand.php?brenda_ligand_id=19832) | |
| CYP 76C1 like | Geraniol 8-hydroxylase | **-26.24** | [geraniol](https://www.brenda-enzymes.org/ligand.php?brenda_ligand_id=987) |  |
| CYP 90A1 | [3beta,22alpha-dihydroxysteroid 3-dehydrogenase](https://www.genome.jp/entry/1.14.19.79) | **-56.24** | [6-deoxoteasterone](https://www.genome.jp/entry/C15799) |  |
| CYP 85A1 | Castasterone synthase, brassinoidb6 oxidase | **-49.09** | 6-deoxo-28-norcastasterone |  |
| CYP 89A2 | [Brassinosteroid 6-oxygenase](https://www.genome.jp/entry/1.14.14.179) | **-49.78** | [6-deoxocastasterone](https://www.genome.jp/entry/C15802) |  |
| CYP 71D10 | Premnaspirodiene oxygenase | **-36.09** | solavetivol |  |
|  |  |  |  |  |

Supplementary Table 4: Docking score of interaction between GmCYPs and their substrate.

|  | | |
| --- | --- | --- |
| **miRNA Accession (*Glycine max*)** | **Target gene (GmCYPs)** | **Number of miRNA targeting target gene** |
| gma-miR1514b-5p | CYP71A1 | 3 |
| gma-miR4415a-5p | CYP71A1 |  |
| gma-miR9761 | CYP71A1 |  |
| gma-miR9737 | CYP71AU50 | 2 |
| gma-miR9758 | CYP71AU50 |  |
| gma-miR1512a-3p | CYP71D10 | 4 |
| gma-miR1512a-3p | CYP71D10 |  |
| gma-miR2108a | CYP71D10 |  |
| gma-miR4394 | CYP71D10 |  |
| gma-miR4993 | CYP76C1like | 2 |
| gma-miR4994-5p | CYP76C1like |  |
| gma-miR4403 | CYP78A3 | 4 |
| gma-miR482b-5p | CYP78A3 |  |
| gma-miR482d-5p | CYP78A3 |  |
| gma-miR482e | CYP78A3 |  |
| gma-miR1513a-5p | CYP78A4like | 4 |
| gma-miR1513b | CYP78A4like |  |
| gma-miR1513c | CYP78A4like |  |
| gma-miR4370 | CYP78A4like |  |
| gma-miR1521a | CYP78A5 | 23 |
| gma-miR1535a | CYP78A5 |  |
| gma-miR1535a | CYP78A5 |  |
| gma-miR1535b | CYP78A5 |  |
| gma-miR1535b | CYP78A5 |  |
| gma-miR156c | CYP78A5 |  |
| gma-miR156d | CYP78A5 |  |
| gma-miR156e | CYP78A5 |  |
| gma-miR156i | CYP78A5 |  |
| gma-miR156j | CYP78A5 |  |
| gma-miR156l | CYP78A5 |  |
| gma-miR156m | CYP78A5 |  |
| gma-miR393h | CYP78A5 |  |
| gma-miR393i | CYP78A5 |  |
| gma-miR393j | CYP78A5 |  |
| gma-miR393k | CYP78A5 |  |
| gma-miR4994-3p | CYP78A5 |  |
| gma-miR5377 | CYP78A5 |  |
| gma-miR9729 | CYP78A5 |  |
| gma-miR9734 | CYP78A5 |  |
| gma-miR9742 | CYP78A5 |  |
| gma-miR9760 | CYP78A5 |  |
| gma-miR9766 | CYP78A5 |  |
| gma-miR5043 | CYP78A5like | 4 |
| gma-miR5043 | CYP78A5like |  |
| gma-miR5782 | CYP78A5like |  |
| gma-miR9761 | CYP78A5like |  |
| gma-miR1513c | CYP78A6like | 7 |
| gma-miR166h-5p | CYP78A6like |  |
| gma-miR166j-5p | CYP78A6like |  |
| gma-miR4403 | CYP78A6like |  |
| gma-miR482b-5p | CYP78A6like |  |
| gma-miR482d-5p | CYP78A6like |  |
| gma-miR482e | CYP78A6like |  |
| gma-miR4382 | CYP82A2 | 3 |
| gma-miR5771 | CYP82A2 |  |
| gma-miR9742 | CYP82A2 |  |
| gma-miR1510a-3p | CYP85A1 | 3 |
| gma-miR172k | CYP85A1 |  |
| gma-miR5677 | CYP85A1 |  |
| gma-miR166m | CYP89A2 | 3 |
| gma-miR3522 | CYP89A2 |  |
| gma-miR4353 | CYP89A2 |  |
| gma-miR156a | CYP90A1 | 11 |
| gma-miR156f | CYP90A1 |  |
| gma-miR156h | CYP90A1 |  |
| gma-miR156q | CYP90A1 |  |
| gma-miR156s | CYP90A1 |  |
| gma-miR156u | CYP90A1 |  |
| gma-miR156v | CYP90A1 |  |
| gma-miR156w | CYP90A1 |  |
| gma-miR156x | CYP90A1 |  |
| gma-miR156y | CYP90A1 |  |
| gma-miR171a | CYP90A1 |  |
| gma-miR1512a-3p | CYP93A3 | 9 |
| gma-miR167i | CYP93A3 |  |
| gma-miR169b | CYP93A3 |  |
| gma-miR169c | CYP93A3 |  |
| gma-miR169e | CYP93A3 |  |
| gma-miR169u | CYP93A3 |  |
| gma-miR169v | CYP93A3 |  |
| gma-miR5041-5p | CYP93A3 |  |
| gma-miR9752 | CYP93A3 |  |
| gma-miR166a-5p | CYP98A2 | 7 |
| gma-miR166c-5p | CYP98A2 |  |
| gma-miR166l | CYP98A2 |  |
| gma-miR171b-3p | CYP98A2 |  |
| gma-miR171r | CYP98A2 |  |
| gma-miR171s | CYP98A2 |  |
| gma-miR4389 | CYP98A2 |  |
| gma-miR166a-5p | CYP98A2like | 5 |
| gma-miR166c-5p | CYP98A2like |  |
| gma-miR166l | CYP98A2like |  |
| gma-miR5038a | CYP98A2like |  |
| gma-miR5038b | CYP98A2like |  |

**Supplementary Table 5: In-silico analysis of miRNA target site in GmCYPs-transcript of *G. max***
